# Genomic and proteomic characterization of a newly isolated *Paenarthrobacter ilicis* strain and its plasmid-mediated xanthan degradation

**DOI:** 10.1128/spectrum.01690-25

**Published:** 2025-12-10

**Authors:** Michael Thomas, Andreas Schlüter, Jenny Fjodorova, Christian Rückert, Tobias Busche, Karsten Niehaus

**Affiliations:** 1 Department of Proteome and Metabolome Research, Faculty of Biology, Bielefeld University 98894, Bielefeld, Germany; 2 Center for Biotechnology Bielefeld (CeBiTec), Bielefeld University 9167, Bielefeld, Germany; 3 Jungbunzlauer AG 695258 , Ladenburg, Germany; 4 Medical School OWL, Bielefeld University 117229, Bielefeld, Germany; Institute of Parasitology, Biology Centre, ASCR, Ceske Budejovice, Czech Republic

**Keywords:** xanthan degradation, *Paenarthrobacter*, proteomics, proteome, plasmid

## Abstract

Environmental soil samples enriched with xanthan gum led to the discovery and isolation of a novel strain of motile *Paenarthrobacter ilicis* in Bielefeld, Germany, which possesses a previously uncharacterized xanthan utilization gene region on its sole plasmid. This bacterium is the first member of the *Paenarthrobacter* genus to feature the ability to degrade xanthan. Growth experiments elucidated the optimal growth conditions with regard to pH (7.0) and temperature (28°C–30°C). The genome of *Paenarthrobacter ilicis* strain 6C consists of two replicons, namely a chromosome with 4,049,144 nucleotides (62.8% GC content) and a plasmid of 147,742 base pairs (61.8% GC content). This novel *Paenarthrobacter ilicis* strain carries 3,806 predicted coding regions, approximately half (51%) of which were verified with proteome analysis. Cultures grown with either glucose or xanthan as a carbon source were compared in relation to protein abundances at four growth stages and time points with regard to both intracellular and extracellular protein fractions. The proteome analysis (data are available via ProteomeXchange with identifier PXD063987) was undertaken using an LC-ESI-MS-MS shotgun proteomics approach, and the proteins were identified and quantified via label-free quantification using MaxQuant 2.6.6. This proteomics investigation exposed significant differences in protein abundances with regard to time and condition. Among the most highly enriched proteins seen when feeding exclusively on xanthan as a carbon source were enzymes encoded on a xanthan degradation plasmid (pPANIL_6C) with a xanthan utilization region coding for the PL8 xanthan lyase, GH38-, GH3-, GH9-family proteins, and several sugar transporters. These proteins constitute key components of a prospective xanthan degradation pathway.

**IMPORTANCE:**

A novel *Paenarthrobacter* isolate was sequenced and characterized by proteome analysis to provide the first clear look at a novel genus in the realm of xanthan-degrading microorganisms. This research provides additional groundwork for the ongoing characterization of *Paenarthrobacter*, as well as widening the understanding of xanthan-degrading microorganisms. For the first time, a xanthan degradation region was identified on a plasmid 1 kb directly downstream from a mobilization gene (*mobF*), posing the question of whether this metabolic capacity can be shared through horizontal gene transfer. Overall, this research expands the current knowledge base regarding *Paenarthrobacter* biology, as well as microbial xanthan degradation and utilization.

## INTRODUCTION

Extracellular polysaccharides play a major role in mediating bacterial interactions with their environment and exhibit great diversity in their structure and biological activity (1). A prominent role of these exopolysaccharides is to provide bacteria with increased protection from abiotic stressors such as drying (2, 3). The varying structure and composition of extracellular bacterial polysaccharides can induce diverse biotic interactions, ranging from the induction of symbiotic root nodules as seen with *Rhizobia* species (4, 5) to the pathogenic infection of plants as with the gram-negative *Xanthomonas campestris* (6, 7).

**Fig 1:**
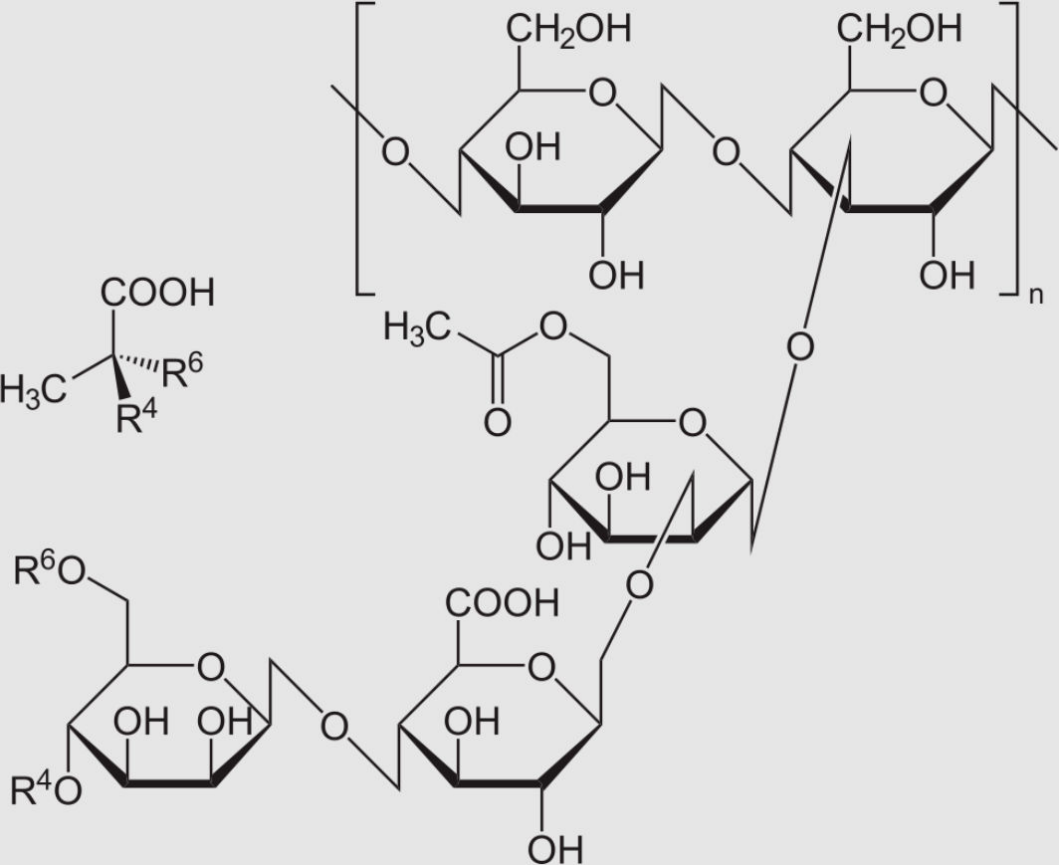
Xanthan consists of a β-(1→4)-D-glucan backbone (cellulose-like) bearing, at O-3 of alternate glucose residues, a trisaccharide side chain Manp-(1→4)-GlcA-(1→2)-Manp. The terminal mannose is frequently pyruvylated as a 4,6-O-(1-carboxyethylidene) ketal; R⁴ and R⁶ mark the two hydroxyls (O-4 and O-6) that participate in this ketal linkage. The inner mannose can be O-acetylated (acetyl group shown). *n* denotes the number of repeating units along the polymer chain (structure adapted from the Wikipedia entry “Xanthan gum”).

The phytopathogenic *Xanthomonas campestris* subdues the host plant’s pathogen response system, primarily *Brassicaceae*, through the secretion of the polysaccharide xanthan (Fig. 1). Xanthan is composed of a *beta-*1,4-linked glucose backbone linked to a side chain starting with a mannose, which is bound to glucuronic acid, and then linked to a terminal mannose. This increases susceptibility to infection when secreted (8, 9). Interestingly, the structure of xanthan has effects on its biological effects. For example, a truncated polysaccharide structure failed to suppress host immunity (8, 10). Therefore, the specific structure of the polysaccharide mediates its biological activity.

The polysaccharide xanthan is also one of the most economically relevant bacterial polysaccharides, with uses ranging from the petroleum industry (11) to pharmaceutical use (12). Moreover, it is used as a common food additive with a robust safety profile and no maximal dose limit (13). Indeed, the specific structure of the xanthan chains within the polysaccharide can impact its rheological effects, and changes in molecular structure can have variable impacts that are not always linearly related to molecular size (14, 15).

The specific decoration of xanthan side chains within the polysaccharide structure directly influences its rheological properties. Consequently, the ability to modify xanthan’s molecular composition—and thereby tailor its physical characteristics—holds considerable industrial relevance, as structural alterations translate into changes in its functional behavior (15). Therefore, enzymes that are responsible for altering the xanthan structure are sought, identified, and then analyzed to provide information on substrate specificity and activity, as demonstrated with the xanthan lyase PL8 of *Paenibacillus nannanis* (16). So far, existing protein structures for xanthan-degrading enzymes have come primarily from *Bacillus* and *Paenibacillus* (17), as well as *Microbacterium* genera (18), and most recently, *Bacteroides* and *Ruminococcus* (19). Given the relatively small selection of characterized xanthan degradation pathways and enzymes, novel environments are likely to uncover more genera, pathways, and enzymes, thereby enhancing our understanding of the specific structural correlates regarding their activity on the xanthan polysaccharide. In this study, a novel *Paenarthrobacter* strain was isolated that is able to use the xanthan polysaccharide as a sole carbon source. Most interestingly, the genes coding for enzymes for xanthan degradation appear to be located on a plasmid. This offers new possibilities for altering xanthan structure and understanding xanthan degradation.

## RESULTS

### Isolation of xanthan-degrading strains and initial physiological characterization

The soil isolate that showed the most promising xanthan degradation came from a replicate of soil from a cabbage patch. Following inoculation in M9 culture media with 0.5% xanthan as a sole carbon source, this soil isolate grew to an OD_600_ of 4.0 over 96 h and was able to reduce the viscosity to below measurable levels compared to the medium when measured in the Rheotec Viscometer. This isolate was then diluted to 10^−3^ in PBS at pH 7.0 and applied to M9 culture media with 0.5% xanthan as the sole carbon source on agar plates for serial single colony streaking.

Following three rounds of single colony streaks on agar with M9 media containing 0.5% xanthan as the sole carbon source, the yellow colony-producing strain was regrown in M9 media with 0.5% glucose as the sole carbon source and harvested as described in the proteomics experiment. Based on this isolate being the sixth replicate obtained from the cabbage soil, it was given the isolate designation 6C going forward.

### Monitoring viscosity changes using sterile culture supernatant

**Fig 2:**
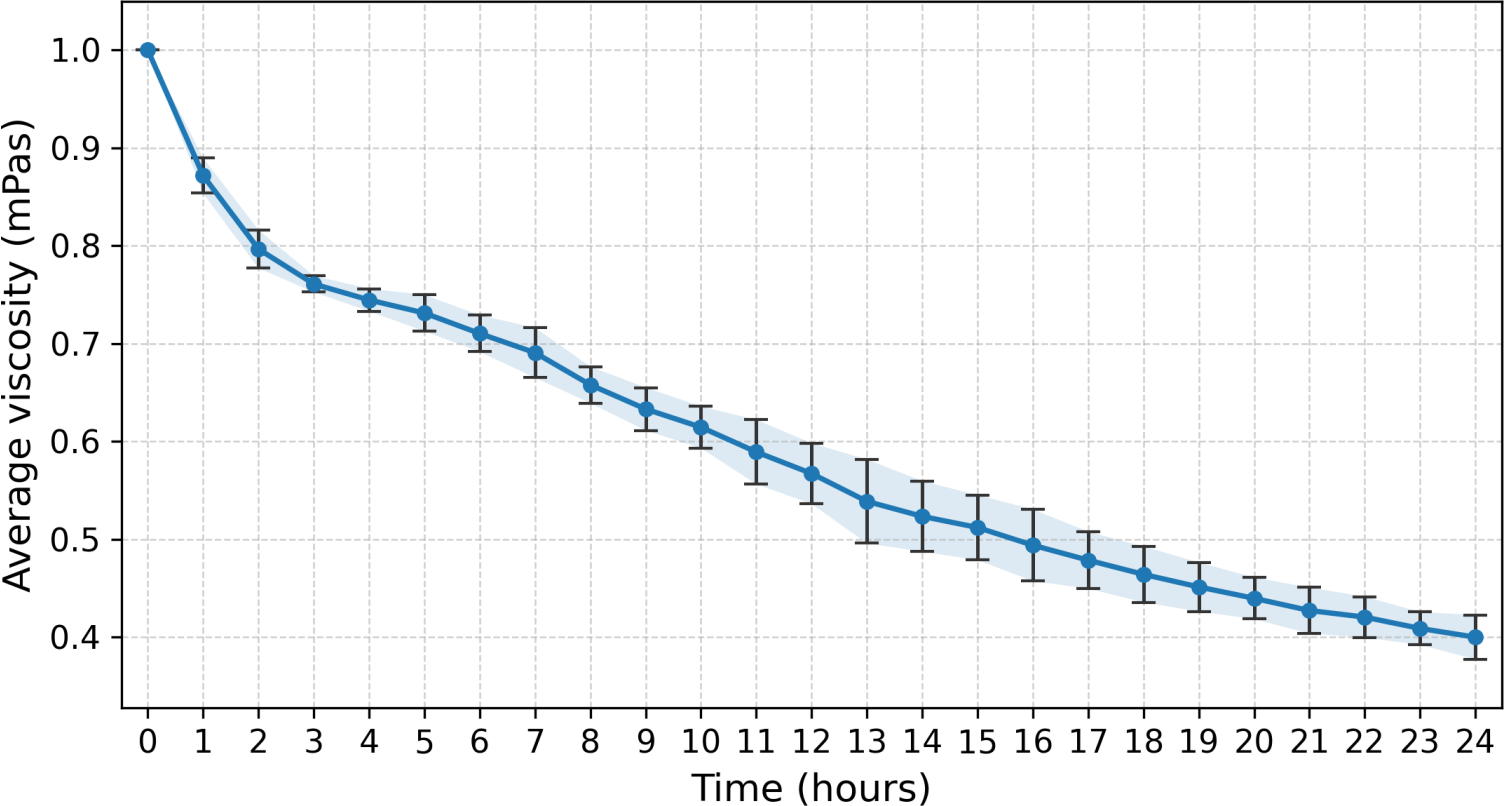
Hourly relative viscosity of a 1% xanthan solution with 1× M9 salts and 200 µL of sterile filtered culture supernatant given in reference to the initial viscosity (defined in millipascals) was measured for 24 h with a Rheotec Viscometer in each experiment (*n* = 3).

After the initial isolation of the *Paenarthrobacter ilicis strain 6C*, its xanthan degradation capacity was investigated using a 1–50 dilution of the sterile filtered culture media from the 48 h cultivation point (OD_600_ = 1.8) in a 1% solution of xanthan. The sterile filtered supernatant was added to a 1% xanthan solution containing the standard concentration of M9 salts at one rotation per minute in the Rheotec Viscometer, and viscosity was measured hourly. As can be seen in Fig. 2, based on three separate experimental runs, the initial viscosity appears to be reliably reduced by approximately 20% within 2 h at 34°C, whereby the slope of change then reduces and appears to go down linearly to 40% of the initial viscosity by 24 h, at which point it stabilizes between 35% and 38% by 48 h (data provided in Data Sheet S5).

### Determination of isolate 6C growth parameters

**Fig 3:**
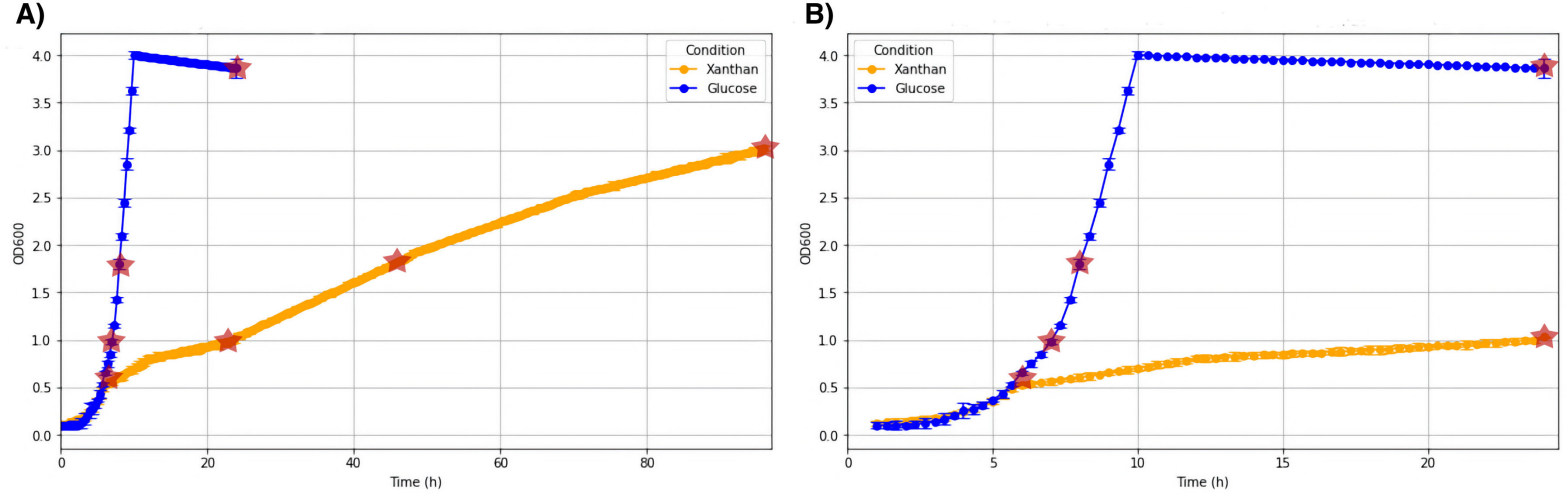
Growth of *Paenarthrobacter ilicis strain 6C* in M9 medium containing either 0.3% (wt/vol) xanthan (orange) or 0.3% (wt/vol) glucose (blue). Proteomic sampling points are denoted with a red star, with panel **A** showing growth for 24 h to highlight growth in glucose M9 media and panel **B** showing the full 96 h to show the growth in xanthan M9 media.

Cell density was measured photometrically based on the absorption of light at 600 nm in M9 containing glucose or xanthan as sole carbon sources as defined in Materials and Methods. Initial growth rates during the first 6 h were comparable between xanthan and glucose media, at which point the glucose samples maintained an exponential growth and the xanthan cultures followed a more linear growth rate. These data can be seen in Fig. 3, where panel A shows the growth over 24 h for xanthan and 24 h for glucose, and panel B shows the growth for 96 h for xanthan and 24 h for glucose. These differences are necessary to show the growth under both conditions in detail.

**Fig 4:**
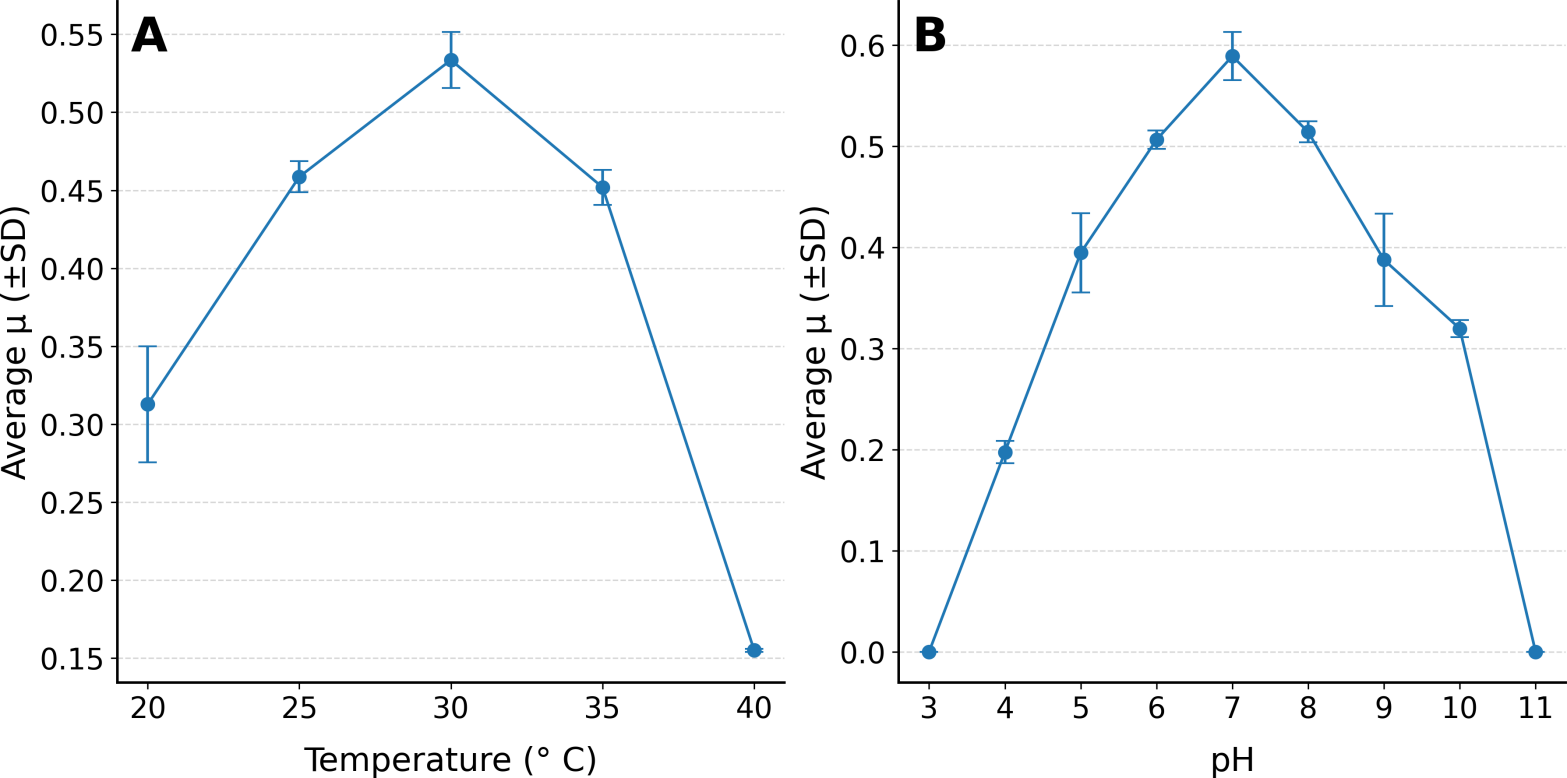
The growth rate resulting from triplicate growth experiments of *Paenarthrobacter ilicis strain 6C* growth in M9 medium with 0.5% glucose as a sole carbon source. The growth rate is expressed as µ. (**A**) The growth rate at temperatures between 20°C and 40°C, and (**B**) the growth rate at pH concentrations ranging from 3 to 11.

Isolate 6C was cultivated at 30°C, pH 7, and shaken at 180 rpm as a control condition for obtaining physiological optima. Growth was observed at temperatures between 20°C and 35°C and at pH 4–10 (Fig. 4A and B).

The maximal growth rate was approximately 0.54 µ to 0.55 ± 0.015 μ (growth rate µ), with the highest growth rate seen at 30°C and at pH 7.

According to the EnteroPluri test, isolate 6C was unable to anaerobically degrade glucose, as there was only a slight orange hue, although gas was produced, showing glucose utilization. While there was no lysine or ornithine decarboxylation, there appeared to be at least some H_2_S and acetoin production. Additionally, although there was no adonitol, lactose, arabinose, or sorbitol utilization, isolate *6C* was able to utilize both urea and citrate. Isolate 6C is a motile (available in the supplemental material), gram-positive, obligate aerobic (available in the supplemental material) bacterium with positive catalase activity.

Sporulation and anabiosis (suspended metabolism that can be seen as deep cellular hibernation) were tested. Isolate 6C was unable to create spore-forming cells. *Bacillus megaterium,* a spore-forming bacterium, provided visual validation of the method. Both *Escherichia coli* and isolate 6C had no cells showing variable coloration.

In order to explore the presence of alternate survival strategies, desiccation experiments were conducted using a limited culture medium that was allowed to dry out completely in the shaker for 72 h. This dried isolate culture could be used to revitalize the culture by adding sterile M9 culture medium, which could then be used for inoculation of new cultures after overnight incubation.

**Fig 5:**
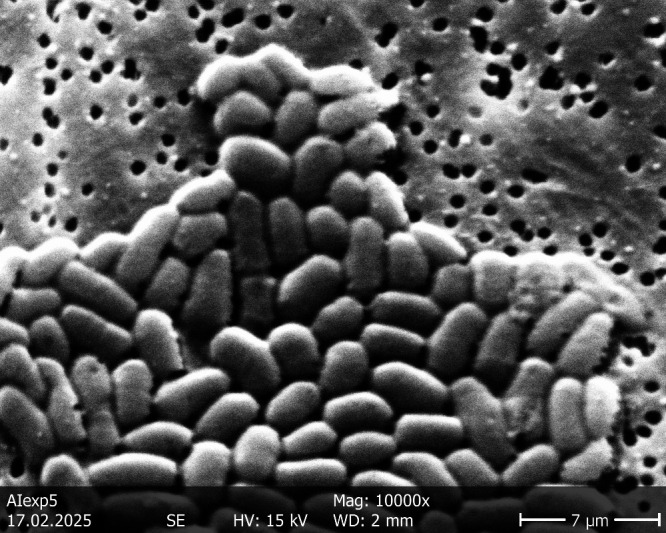
An SEM image of isolate 6C during the exponential growth phase at 10,000× magnification.

The scanning electron microscope (SEM) analysis showed rod-shaped bacteria approximately 2–5 µm long and approximately 1–2 µm wide, as can be seen in Fig. 5.

### Genome sequencing identifies 6C isolate as *Paenarthrobacter ilicis* strain

In order to understand and functionally characterize the 6C isolate, 16s rRNA gene and genomic sequencing were undertaken. Comparison of both the 16s rRNA gene sequences, as well as genomic sequences, identified the 6C isolate as a close relative of *Paenarthrobacter aurescens* and *Paenarthrobacter ilicis,* with approximately 97% identity with either species on the 16S rRNA sequence level.

The genomic sequencing was undertaken using the Oxford Nanopore FLO-MIN112 with MIN-KNOW 22.08.9, resulting in two contigs: a chromosome of 4,049,144 nucleotides (62.8% GC content) and a plasmid of 147,742 bp (61.8% GC content). A total of 3,882 genes were predicted, with 3,806 predicted proteins, of which 1,883 could be functionally annotated (48.8%). Further descriptive statistics of the genome can be seen in Table 1.

**TABLE 1:** Descriptive statistics regarding the *Paenarthrobacter ilicis* strain 6C sequencing and assembly approach

Category	Value
Genome size	4,196,886 bp
Contigs	2
*N*_50_	11,535
Coverage	79-fold
Genes	3,882
CDS (total)	3,806
Genes (coding)	3,760
Genes (RNA)	76
rRNAs (5S, 16S, 23S)	6, 6, 6 (18)
tRNAs	55
ncRNAs	3
Pseudogenes (total)	46

**Fig 6:**
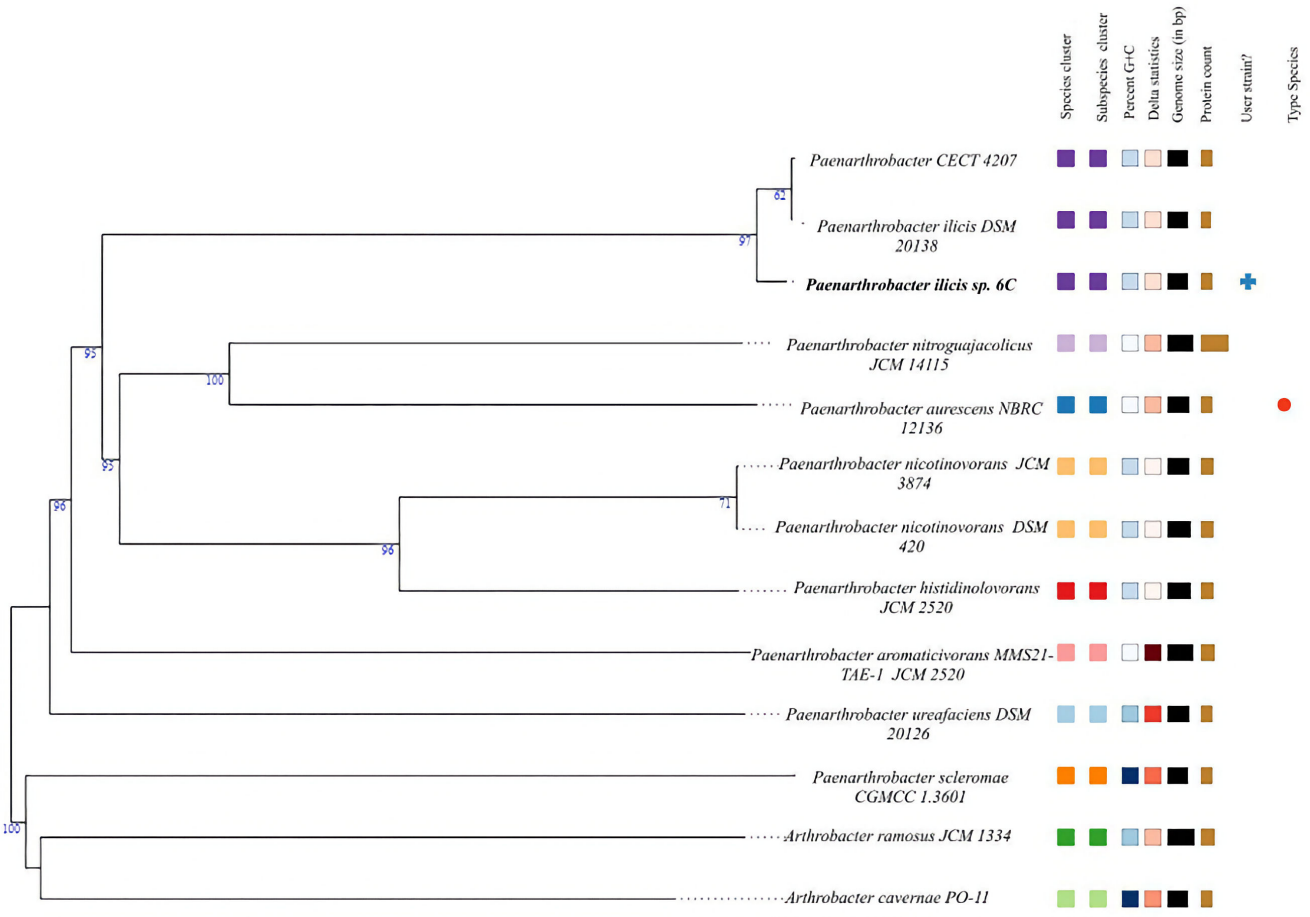
Phylogenetic tree assembled by the TYGS (20) server based on Genome Blast Distance Phylogeny using a GREEDY algorithm. Branches are annotated with the sureness value (0–100). The novel *Paenarthrobacter ilicis strain 6C* is written in bold.

The overall phylogenetic position of the newly discovered *Paenarthrobacter ilicis* strain *6C* was calculated using the GREEDY algorithm of the TYGS server (20), showing an essentially equidistant relationship (97% sureness of clade relationship to 6C but only 67% probability of species-level relationship with each other to 6C) with *Paenarthrobacter ilicis CECT 4207* (average nucleotide identity, ANI = 91%) and *Paenarthrobacter ilicis DSM 20138* (ANI = 92.5%) and TYGS combining them all under the *Paenarthrobacter ilicis* species label (Fig. 6). Further close relatives are *Paenarthrobacter nitroguajacolicus JCM 14115* and *Paenarthrobacter aurescens NBRC 12136*. The isolate 6C was thus renamed and, based on being from the sixth replicate of cabbage soil, was given the designation *Paenarthrobacter ilicis* strain 6C based on the full genome sequence.

The accessory plasmid was analyzed using Edgar 3.0 version 3.2 (21) to compare it to its closest neighbor, *Arthrobacter* sp. FW305 unnamed plasmid CP084565. Of the 141 genes on the *Paenarthrobacter ilicis* strain 6C plasmid, 57 genes are shared between the two according to EDGAR 3.0. They share a type IV secretory system conjugative DNA transfer family protein (GMCHHGAN_003867) with 77.93% identity, alongside MobF (GMCHHGAN_003835) with 79.09% identity. Additionally, 80 genes were found to be unique to the 6C strain. The genes shared on both plasmids showed an average nucleotide identity of 78.88%, which is consistent given the phylogenetic proximity of *Paenarthrobacter* with *Arthrobacter*. Within these 80 unique genes specific to 6C, the entirety of what has been named the “xanthan utilization region” (XUR) (60.2% GC) is found alongside approximately 30 hypothetical proteins.

**Fig 7:**
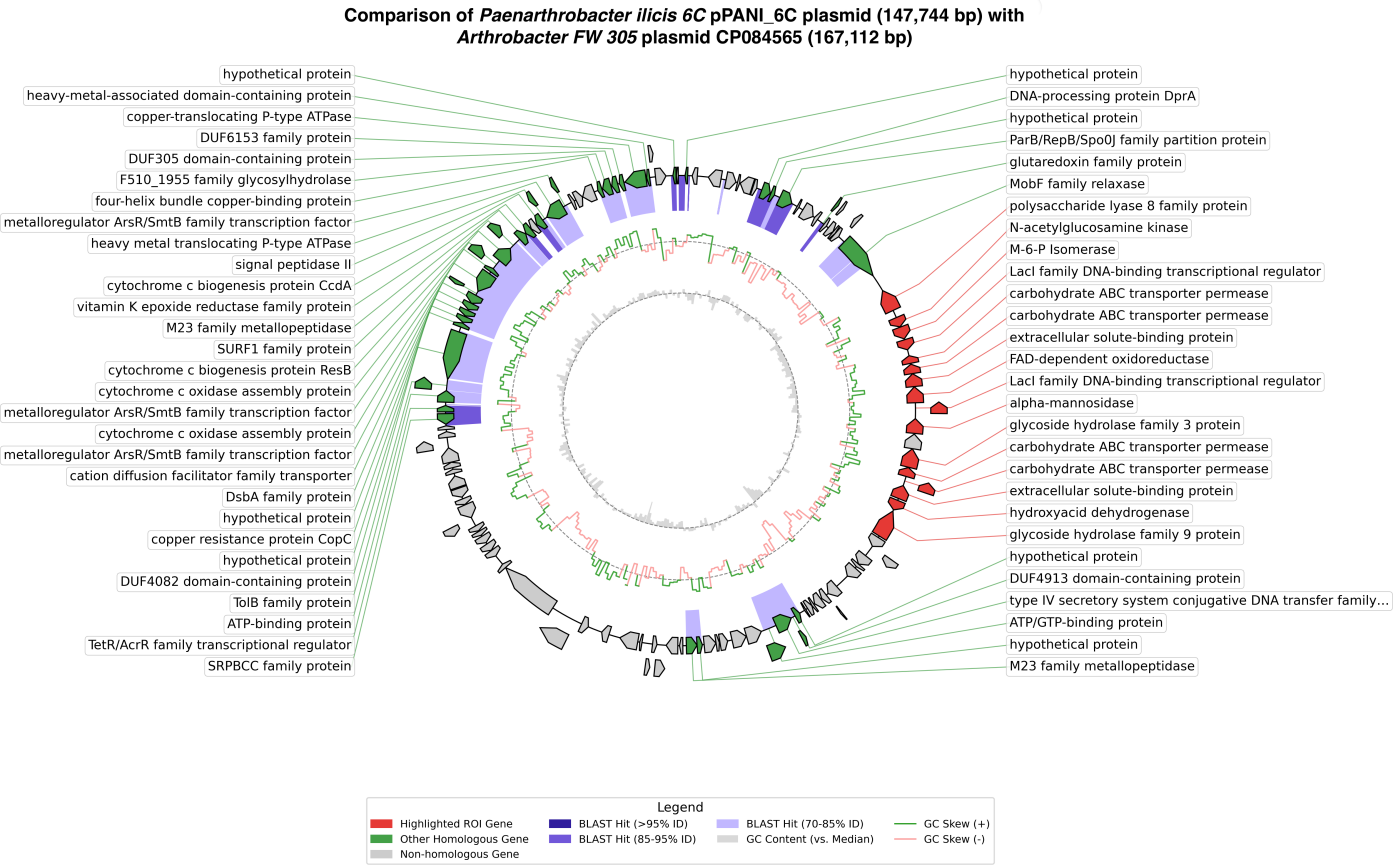
Circular comparison of *Paenarthrobacter ilicis* 6C plasmid pPANIL_6C (GenBank accession No*.* CP173686, 147,744 bp) with *Arthrobacter* sp. FW305-123 (unnamed plasmid, GenBank accession No. CP084565*,* 167,112 bp). BLASTn similarities are indicated by colored regions on the innermost ring. The outer ring shows annotated genes as arrows (orientation = transcriptional direction). The arrow indicates the direction of transcription. The (region of interest, ROI) genes are highlighted in red, and additional genes with significant homology (>80%) to CP084565 are green and given in Data Sheet S6; all other genes are colored gray. CDSs that overlap with downstream or upstream CDSs are shown on the outermost circle of the plasmid map to clearly represent their length and orientation. A single gray arrow inside the red ROI block marks a predicted pseudogene. Side labels summarize the best functional annotation with coloring implying identity overlap. Inside the ring, the filled gray trace shows GC content relative to the sequence median, and the overlaid lines show GC skew ([G − C]/[G + C]; green, positive; salmon, negative).

A visualization of the CP084565 plasmid with the pANIL_6C plasmid at 80% identity shows that the homologous genes are clustered in a region, and that the XUR is fully unique to the *Paenarthrobacter ilicis* sp*. 6C* pANIL6C plasmid. There appears to be a clear negative GC-skew within the region versus the rest of the plasmid (Fig. 7).

**Fig 8:**
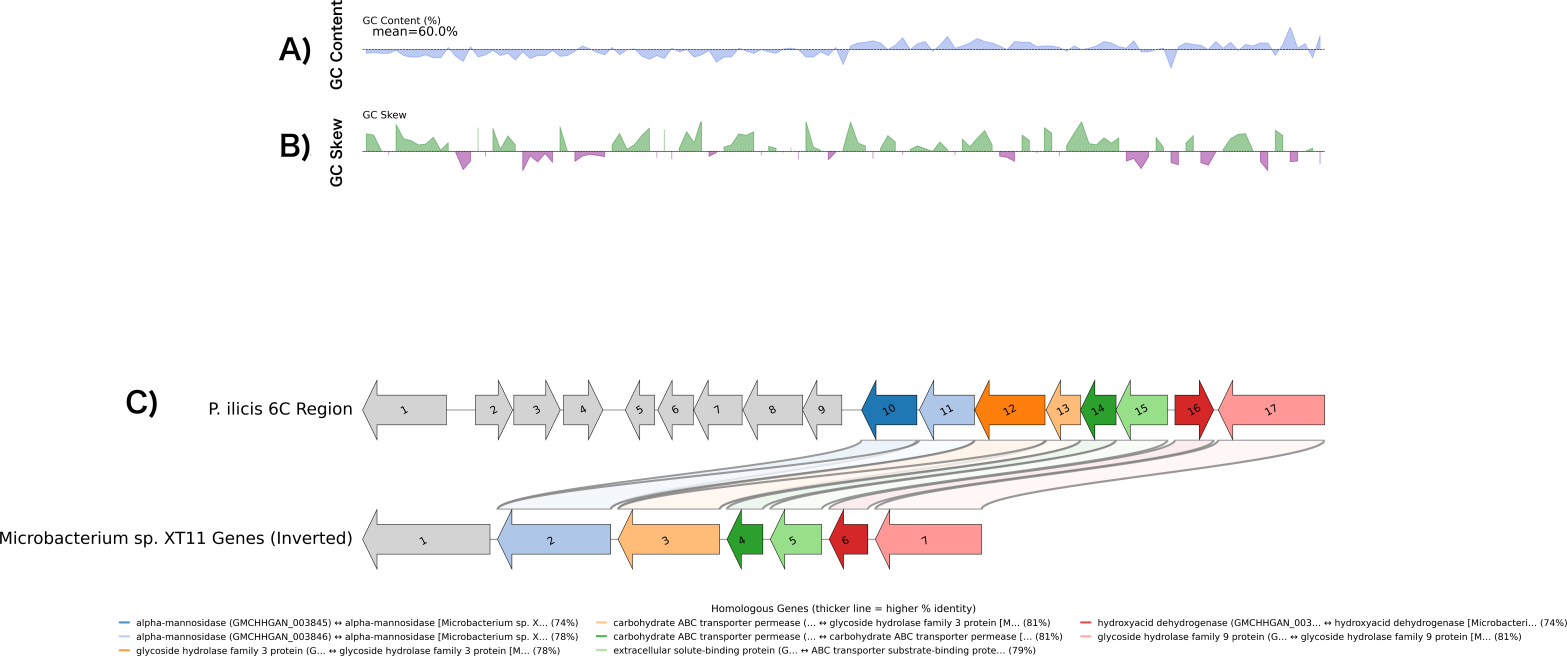
GC landscape and gene synteny of the XUR locus in *Paenarthrobacter ilicis* 6C and the cognate region in *Microbacterium* sp. XT11. (**A**) GC content (%) across the *P. ilicis 6C* XUR region shown as a sliding-window profile. The dashed horizontal line marks the regional mean GC content (60%). (**B**) GC skew, calculated as (G − C)/(G + C), for the same interval. Positive skew values (green) and negative values (purple) indicate local enrichment of G or C, respectively. (**C**) Gene-level synteny between the *P. ilicis* 6C segment (top; open reading frames PAI1-PAI17) and the corresponding XT11 locus (bottom; XT1-XT7, drawn inverted to maximize collinearity). Arrows denote coding sequences, with arrow direction indicating transcriptional orientation; gray arrows mark genes without an annotated counterpart in the other genome. Colored ribbons connect putative homologs and ribbon thickness scales with pairwise amino-acid identity. The core collinear block includes two α-mannosidases (PAI10/PAI11 ↔ XT2), a glycoside hydrolase family 3 enzyme (PAI12 ↔ XT3), ABC-transporter components (PAI13-PAI15 ↔ XT4-XT5), a hydroxyacid dehydrogenase (PAI16 ↔ XT6), and a glycoside hydrolase family 9 protein (PAI17 ↔ XT7), with identities in the ~74%–81% range as indicated in the legend. For reference, the *P. ilicis* ORFs from left to right are PAI1 PL8 polysaccharide lyase (GMCHHGAN_003836), PAI2 N-acetylglucosamine kinase (003837), PAI3 manA (003838), PAI4 LacI-family regulator (003839), PAI5–PAI6 ABC permeases (003840/003841), PAI7 extracellular solute-binding protein (003842), PAI8 FAD-dependent oxidoreductase (003843), PAI9 LacI-family regulator (003844), PAI10–PAI11 α-mannosidase and a copy as a pseudogene (003845 and 003846), PAI12 GH3 (003847), PAI13–PAI14 ABC permeases (003848/003849), PAI15 solute-binding protein (003850), PAI16 hydroxyacid dehydrogenase (003851), and PAI17 GH9 (003852). The XT11 ORFs (left to right on the inverted contig) are XT1 PL8 (AB663_RS05330), XT2 α-mannosidase (AB663_RS05300), XT3 GH3 (AB663_RS05275), XT4 ABC permease (AB663_RS05285), XT5 ABC substrate-binding protein (AB663_RS05295), XT6 hydroxyacid dehydrogenase (AB663_RS05270), and XT7 GH9 (AB663_RS05265). Plots were rendered with pygenomeviewer (pygenomics) (22).

The XUR got this designation due to BLASTp analysis of most of the predicted proteins within this region, matching their highest score to *Microbacterium XT11*’s known xanthan-degrading enzymes. Within the region, *Paenarthrobacter ilicis strain 6C*’s proteins shared high amino acid identities with almost all proteins in the *Microbacterium XT11* xanthan region, ranging from 76% up to 97% for all proteins in the XUR, except for the xanthan lyase PL8 (GMCHHGAN_003836), where there was only 40% amino acid identity and 35.7% DNA identity (tables containing the amino acid and nucleotide identities between the XUR and *Microbacterium XT11* can be found in supplemental data sheets). Interestingly, the order and orientation of the genes found within this region are also identical to that found in *Microbacterium XT11* (18). Therefore, the primary functional genes in the region are homologous with the exception of the xanthan lyase protein, as demonstrated by a BLASTn analysis and alignment (Fig. 8).

The full protein and DNA identities are given for positive matches (Table 2) with the full BLASTn and BLASTp tables given in the supplemental data sheets.

**TABLE 2:** Comparison of *Microbacterium XT11* analogs to *Paenarthrobacter ilicis* 6C proteins at the DNA and amino acid levels

Protein	Accession for *P. ilicis* 6C	Accession for MXT11 protein	AA% identity	DNA identity (%)	Query coverage (%)
Xanthan lyase (PL8)	3836	WP_067196463.1	40.05	35.75	100.00
Endoxanthanase GH9	3852	WP_198147935.1	86.75	80.59	100.00
Transporter (ABC permease)	3840				
Transporter (ABC permease)	3841	WP_067196455.1	34.71		98.00
Transporter (ABC permease)	3848	WP_067196455.1		81.11	99.00
Transporter (ABC permease)	3849	WP_067196455.1	84.11	81.13	100.00
Solute binding protein	3842				
Solute binding protein	3850	WP_067196453.1	85.44	79.26	100.00
GH3	3847	WP_067196459.1	82.11	77.74	98.00
GH38 α-mannosidase	3845	WP_067196461.1	75.50	74.00	99.00
GH38 α-mannosidase (pseudogene)	3846	WP_067196461.1	77.53	78.06	64.00
Hydroxyacid dehydrogenase	3851	WP_198147936.1	76.01	73.56	100.00
N-acetylglucosamine kinase	3837				
Mannose-6-phosphate isomerase (manA)	3838				
LacI-family regulator	3839				
LacI-family regulator	3844				
FAD-dependent oxidoreductase	3843				

**Fig 9:**
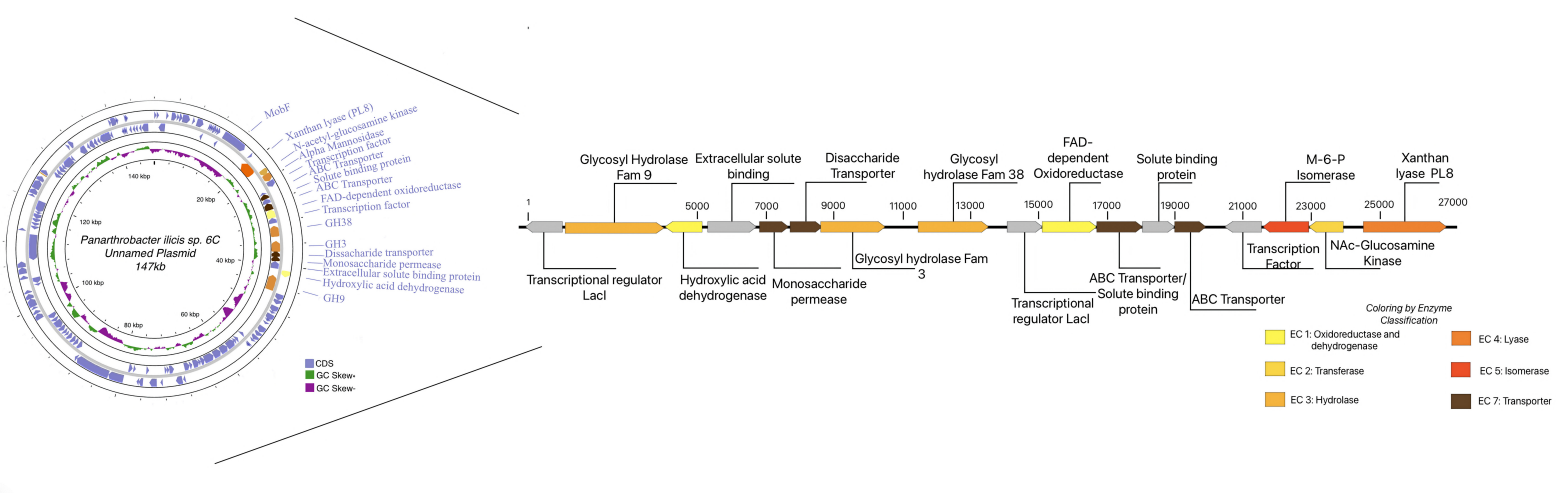
Modified Proksee (23) readout of the *Paenarthrobacter ilicis strain 6C* plasmid pPANIL_6C with 141 genes and a sequence length of 147 kb. The genes outside the xanthan degradation/utilization region are not marked aside from *mobF* to provide a clearer focus on the unique xanthan utilization region. The region is shown in higher resolution and colored by EC number, whereby proteins lacking an EC number or sequence-derived function are shaded gray. Oxidoreductases and dehydrogenases are light yellow, transferases are light orange, hydrolases are orange, lyases are dark orange, isomerases are red, and transporters are brown.

The xanthan utilization region and adjacent genes are shown in Fig. 9, where only the XUR is annotated and classified functionally using a color spectrum related to the EC numbers (Enzyme Classification Number). Proteins lacking a classification are gray, and EC number color codes are given relative to a yellow-brown spectrum. Interestingly, the genes within this region have a GC ratio of 50%–60%, which is lower than in the rest of the *Paenarthrobacter ilicis* strain 6C genome (62.8%) and the plasmid (61.8%) on which it is located.

Additionally, the regional GC-enrichment analysis of the full XUR showed a significant difference and decrease relative to the host (*Paenarthrobacter ilicis* sp. 6C*,* 62.76%) of the XUR (−2.77%), which is supported by both binomial and permutation tests (*P* = 0.02683). The regional difference was within the plasmid-wide variation (*P* = 0.035).

The trimmed genomic data are available from SRA via BioProject PRJNA1182270. The annotated chromosome and plasmid are available under the GenBank accession numbers CP173685 and CP173686.

### Differences in the *Paenarthrobacter ilicis* 6C proteome grown in medium with xanthan relative to glucose

In order to better understand the cellular contexts and enzymatic machinery involved in xanthan degradation and utilization, a comparative proteomics experiment was undertaken in which *P. ilicis* 6C was cultivated in M9 medium containing either 0.3% (wt/vol) glucose or xanthan as the sole carbon source. In the proteomics experiment, 1,956 proteins were identified, with approximately 1,900 proteins identified for each carbon source condition. This is approximately 51% of the entire predicted proteome.

The comparative proteomics experiments analyzed the differences between the two carbon source conditions in both intra- and extracellular compartments at four different optical densities during the growth of the strain, which can be seen denoted by the red stars in the growth charts (Fig. 3). For the sake of clarity, we will focus on the results as they relate primarily to the proteins coded by genes found within the xanthan utilization region.

**Fig 10:**
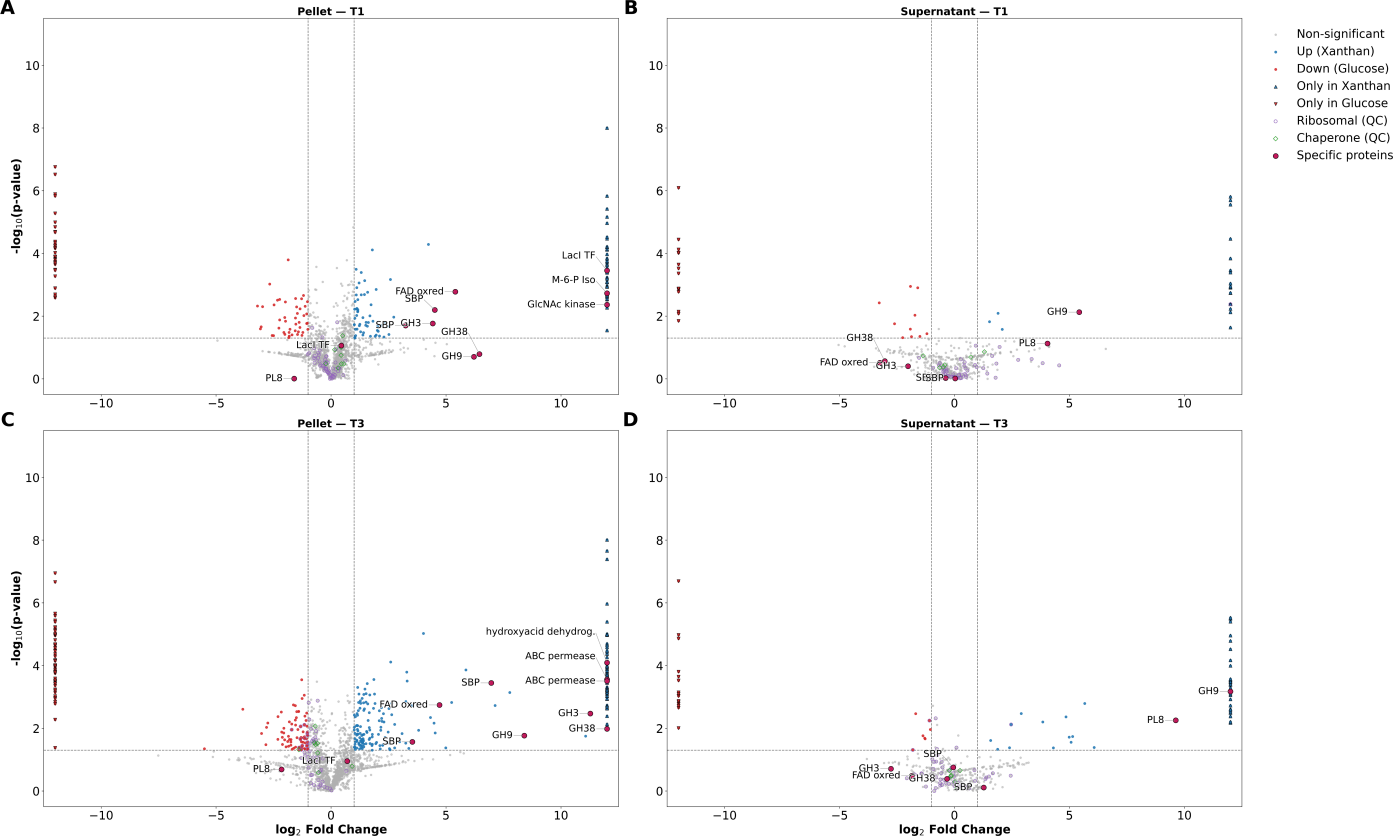
Relative protein abundance and significance are shown in panels A–D at the OD_600_ of 0.6 (time point 1) and 1.8 (time point 3). A maximum was arbitrarily set at 6 for the Log2 fold change for visualization purposes. Panels **A** and **B** show intracellular abundances and significance, whereas panels **C** and **D** show data from the supernatant. Log2 fold differences in protein abundance are shown on the *X*-axis, whereas significance is displayed on the *Y*-axis. Proteins found within the plasmid for the xanthan utilization region are highlighted as outlined maroon dots in panels **A–D**. Protein names are abbreviated to improve figure clarity.

The growth periods with the most pronounced differences in protein abundance were the first time point (OD = 0.6) and the third time point (OD = 1.8). Between these two growth points, we saw an increase in the proportion of proteins encoded within the XUR that are significantly overabundant and an increase in the relative significance (Fig. 10).

**Fig 11:**
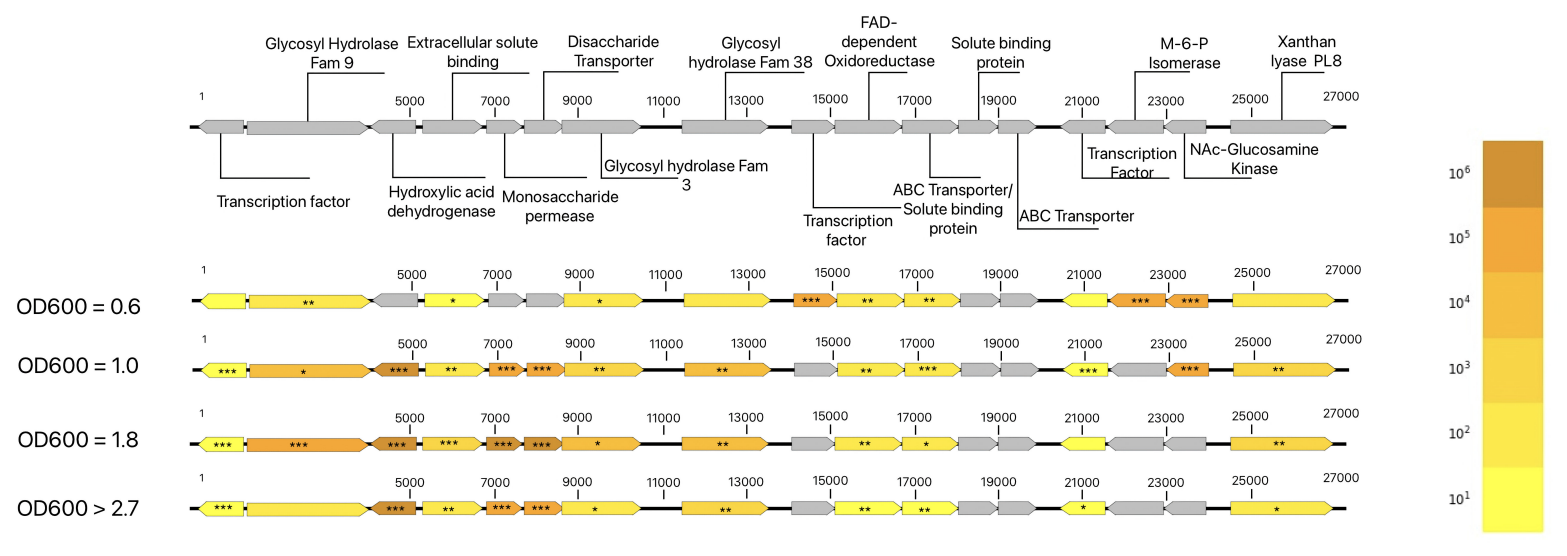
Relative abundance of proteins encoded in the XUR of the plasmid of *P. ilicis* 6C when cultivated with xanthan versus glucose as a sole carbon source. In addition, the effect of different growth phases is shown. The relative protein abundance for *P. ilicis* strain 6C is shown at four time points for proteins encoded within the XUR, with significance based on heteroscedastic *t*-tests using biological replicates. Relative protein abundance is given with regard to the power of 10 using the color scale seen on the right. Stars are used to connote significance with * being equivalent to *P* < 0.05, ** being equivalent to *P* < 0.01, and *** being equivalent to *P* < 0.001.

There are many other proteins that are either over- or underabundant in the xanthan condition relative to the glucose condition at each time point, and with over 1,800 proteins measured intracellularly at each time point, the focus remains on the xanthan utilization region. The relative overabundance in the xanthan condition, as well as the significance based on a homoscedastic *t*-test, was calculated. The fold differences and relative significance can be seen in Fig. 11.

At an OD_600_ of 0.6, out of 15 proteins measured in the xanthan utilization region, 8 were significantly overabundant in the xanthan condition compared to the glucose condition (53.3%). At an OD_600_ of 1.0, 12 proteins were measured, and all of them were significantly overabundant in the xanthan condition (100%). At an OD_600_ of 1.8, out of 12 proteins measured, 10 were significantly overabundant (83.3%).

At an OD_600_ of greater than 2.7, 12 proteins were measured, with 11 (91.6%) showing significant overabundance in the xanthan condition.

A gene enrichment analysis was undertaken to reaffirm the findings for the individual proteins, showing very high significance at every time point. The specific values can be seen in Table 3.

**TABLE 3:** The results of a gene enrichment analysis, with the time points given relative to the number of significant genes in the XUR ROI (region of interest) using pellet LFQ intensities for all proteins except for the xanthan lyase PL8 and GH9 (which were taken from the supernatant)

Optical density (600 nm)	Proteins detected (ROI)	Proteins detected (genome)	Mean log2fc (ROI)	Mean log2fc (genome)	K-S-test *P*-value	*t*-test *P*-value
0.6	11	1,682	5.208	0.1056	2.77E-06	0.000017
1.2	12	1,764	8.56	0.5357	1.92E-06	0.000018
1.8	11	1,778	9.254	0.4498	2.77E-05	0.000096
>2.7	11	1,741	6.902	0.1841	2.77E-05	0.000134

In summary, as the cultures grow and progress to higher optical densities, a majority of the proteins encoded in the putative xanthan utilization region are significantly overabundant in the xanthan condition compared to the glucose condition. This tendency was significant at every time point and rose from 53.3% of detected proteins at the first growth phase being measured as significantly overabundant in the xanthan condition at OD 0.6, with this number peaking at 100% at an OD_600_ of 1.0. The growth then ended with 91.6% of detected proteins encoded and detected within the XUR being significantly overabundant at OD_600_ greater than 2.7. The overabundance of the majority of proteins in this region in the xanthan condition is observed at each of the four sampling points measured, with the lowest relative proportion of proteins significantly overabundant seen at an OD_600_ of 0.6, but the region remains highly significant at all points.

## DISCUSSION

### Physiological properties of *Paenarthrobacter ilicis* strain 6C indicate that the species is a mesophilic xanthan degrader

The strain isolation strategy yielded a novel genus of xanthan degradation, namely *Paenarthrobacter*. Although *Paenarthrobacter* and *Arthrobacter* are known for degrading various organic products such as diesel (24), organic pollutants (25), or pharmaceutical compounds (26), this is the first instance of xanthan degradation within the *Paenarthrobacter/Arthrobacter* genus.

The results of the physiological characterization experiments, namely the temperature and pH optima experiments, showed an optimum growth rate at pH 7.0 and 28°C–30°C. The motile *Paenarthrobacter ilicis* strain 6C shares a similar physiological profile to the overarching *Paenarthrobacter ilicis* species based on a comparison with *Paenarthrobacter ilicis* sp*. CR2* (BacID 7619) and *Paenarthrobacter aromaticivorans* sp*. Nov* (26). This enables the reasonable completion of the missing portion of the temperature curve (Fig. 4), based on existing species-level knowledge. This means the *Paenarthrobacter ilicis* strain 6C can likely grow at temperatures between 10°C and 35°C, with the upper echelon slightly lower in our experiments compared to *Paenarthrobacter ilicis Cr-2,* which can still tolerate 40°C and *Paenarthrobacter ilicis aromaticivorans* to 37°C (26). Additionally, we have established growth between pH 4 and 10 for the species, showing a similar range to that of the other *Paenarthrobacter* species. The main contradiction in our physiological data to *Paenarthrobacter ilicis Cr-2* or *aromaticivorans* findings is that *6C* is motile. This was established both genomically, based on the identification of 29 flagellar chromosomally coded proteins (GMCHHGAN_000805 to GMCHHGAN_000839; see Data Sheet S7), and experimentally (see supplemental material). Physiologically, *Paenarthrobacter ilicis 6C* shares a growth optimum of 30°C and pH 7 with the other species level examples, arguing that *Paenarthrobacter* is largely a mesophilic and neutrophilic bacterial genus.

The growth of the 1 × 5 µm-sized *Paenarthrobacter ilicis* strain 6C was multiphasic in xanthan media, as seen in the growth curves. This can be explained by the fact that “cell-free” xanthan was likely not free from the remains of lysed *Xcc* cells and potentially provided easily accessible sugars to boost the growth speed, making it comparable to the glucose condition during the initial growth up until an OD of 0.6 in 0.3% xanthan M9 medium. This will be elaborated on more in-depth in the proteomics section of Discussion, where the chronological resolution allows for a better understanding of the growth phases.

Interestingly, *Paenarthrobacter ilicis* strain 6C appears to show no real overshoot behavior in repeated experiments, attaining essentially the stationary population density and then slowly decreasing from that point, as can be seen in other species from the *Arthrobacter* genus (27, 28). Although *Paenarthrobacter ilicis* strain 6C was unable to form spores and lacks the genes coding for this capacity, it appears to be capable of anabiosis (survival with highly limited metabolism) with revitalization being possible after 72 h of long desiccation and reintroduction of minimal media. This metabolic hyperconservation is established for the *Arthrobacter* genus, which is closely related to *Paenarthrobacter* and represents a viable method for environmental persistence (29). This can help explain the lack of overshoot that *Paenarthrobacter ilicis strain 6C* demonstrates after reaching the stationary phase, with related *Arthrobacter* known for their ability to enter anabiosis when environmental conditions are unfavorable (29).

The ability of *Paenarthrobacter ilicis* strain 6C to grow using xanthan as a sole carbon source was validated, and the ability of the sterile filtered supernatant to reliably reduce viscosity by 60% within 24 h argues for the strain as a new model of xanthan degradation, adding the *Paenarthrobacter* genus to the list of known xanthan-degrading genera.

### Genomic analysis of *Paenarthrobacter ilicis* strain 6C resulted in the identification of a plasmid-bound xanthan degradation region

The approximately 4 Mb chromosome has approximately 62% GC and is comparable to other *Paenarthrobacter* species; for instance, *Paenarthrobacter A01* has a chromosome of 4.88 Mb and 63% GC (30), and *Paenarthrobacter nicotinovorans* has a chromosome of 4.3 Mb and 62% GC as well (30, 31). *Paenarthrobacter ilicis* strain 6C is also clearly clustered with the other *Paenarthrobacter ilicis* species in the TYGS (20) alignment, validating its phylogenetic classification. The most genetically intriguing feature of isolate 6C is the pPANIL_6C 147 kb plasmid, which contains a seemingly unique XUR.

Currently, there are no other examples of a xanthan utilization region lying on a plasmid, which may indicate that this capacity can be horizontally transferred among related species, especially given the close proximity (1 kb) of the *mobF* (GMCHHGAN_003835) gene, which encodes a mobilization protein to the XUR.

Here, the evidence relative to the genetic structure will be discussed in light of the proteomics data, whereas the putative xanthan degradation and utilization pathway will be discussed in the proteomics section of Discussion.

The xanthan degradation region appears to contain all the proteins needed to degrade, import, and initially utilize the xanthan polymer and allows its use as a sole carbon source for growth. The question of whether the region acts as a single genetic element can apparently be answered with a negative: the different proteins are differently abundant at chronologically discrete points and cell densities. Although every protein measured in the region was overabundant in the xanthan condition at the fitting localization at all measuring points, the explicit overabundance and the statistical significance varied, and not every protein was detected at every growth density. For example, the mannose-6-phosphate isomerase (GMCHHGAN_003838) was only detected at an optical density of 0.6 and was highly significant at this time point. It seems likely that the XUR is subject to more complex regulation during growth on xanthan and the concentrations of its degradation products.

Overall, the organization of the XUR appears similar to that of *Microbacterium XT11* (18), including the lack of any GH88 analog. Unlike with *M. XT11*, the XUR for *Paenarthrobacter ilicis* strain 6C is located on the 147 kb large pPANIL_6C plasmid 1 kb downstream from a MobF family relaxase (GMCHHGAN_003835), which is part of the MOB family involved in horizontal gene transfer (32, 33).

These two findings may be interrelated and imply that there may be a common ancestor for the XUR found in both *Paenarthrobacter ilicis* strain 6C and *Microbacterium XT11*, and that *Paenarthrobacter ilicis* strain 6C may hold the first clear indication of a transferable xanthan degradation unit. Interestingly, this gene cluster is expanded relative to the *Microbacterium XT11* region, although it is unclear how many of these additional proteins within the region are essential for xanthan degradation.

Thus, this plasmid-encoded region is likely seeing differential expression relative to transcriptional regulators. Indeed, all transcriptional regulators within the region were detected in the experiment, but they were significantly overabundant at different growth points; at no point were all three significantly overabundant simultaneously. This implies more complex regulation of gene expression relative to stimuli and potentially related to the concentration or detection of relevant metabolites, for instance, through the ABC transporter GMCHHGAN_003842, which is significantly overabundant already at an OD_600_ of 0.6, after which the other transporters (such as GMCHHGAN_003849) become detected and significantly overabundant starting at an OD_600_ of 1.0.

### Proteomic analysis of *Paenarthrobacter ilicis* strain 6C verifies the xanthan degradation region and provides higher resolution of differences in abundance

The comparative proteomics experiment identified approximately 51% of the entire predicted proteome, which is comparable to 53% identified for *Cyanobacterium Synechocystis* (34) or 44.61% identified for lignin degrader *Pandoraea* sp*. ISTKB* (35). The proteomics section will restrict itself to the discussion of proteins found within the approximately 20 kb xanthan degradation region on the 147 kb pPANIL_6C plasmid. The proteomics results will be used to propose a *Paenarthrobacter ilicis* strain 6C putative xanthan degradation pathway and discuss the changes in protein abundance over the span of multiple optical density measurements.

Before the optical density of 0.6, both the xanthan and glucose M9 cultures had grown at roughly the same rate (0.54 μ), and this sample represents the closest measurement chronologically between the two conditions. At this density, the majority of proteins are already overabundant in the XUR (53%), although this is not yet significant for 7 of 15 of the proteins detected within the region, and only at this point was the mannose-6-phosphate isomerase ManA (GMCHHGAN_003838) detected and found to be significantly overabundant. This implies that early growth is potentially not exclusively dependent on xanthan as the sole carbon source, although the terminal mannose is likely already being metabolized before an optical density of 1.0 due to the significance of the mannose-6-phosphate isomerase, GH9, and GH3 in the xanthan condition at an OD_600_ of 0.6. This implies that at an OD_600_ of 0.6, there has been recognition of a limited number of elicitor signals and specific metabolites are being imported, although the majority of metabolic machinery is already significantly overabundant, which increases measurably when compared to all future time points.

At an OD_600_ of 1.0, 13 of the 15 proteins detected within the XUR in this experiment were significantly overabundant at this density. This means that at an OD_600_ of 1.0, 86% of the proteins detected in this region in this experiment are measured as significantly overabundant, including the xanthan lyase PL8, the GH9, the GH38, the GH3, and every transporter detected within the experiment. This is several more in absolute terms and almost twice as many in relative terms than were measured as overabundant at an OD_600_ of 0.6. This implies that substrate-triggered induction of the entire region occurs at some point between the optical density of 0.6 and 1.0. One transcriptional regulator (GMCHHGAN_003844) was only detected and significant at an OD_600_ of 0.6, while the other two (GMCHHGAN_003853 and GMCHHGAN_003839) gained significance going forward.

This implied induction carries over to the optical density of 1.8, where only the “first” transcriptional regulator (GMCHHGAN_003844) loses significance, and the N-acetyl glucosamine kinase (GMCHHGAN_003837) is no longer detected. The results imply that there are small changes in protein expression or degradation that occur between the optical densities of 1.0 and 1.8.

By the final measurement at 24 h for the glucose condition and 96 h for the xanthan conditions, 11 of 15 (73%) of the overall detected proteins within this region over the entire experiment were significantly overabundant. If we look at proteins detected at this time point, 91.6% of the proteins encoded within the XUR remain significantly overabundant at the end in the xanthan condition. Between the optical densities of 1.8 and these measurements at OD_600_ absorptions greater than 2.7, all of the proteins encoded in the XUR remain either equally overabundant or decreased in relative overabundance, implying that the xanthan degradation machinery is still in use but may be finished utilizing certain parts of the polysaccharide and is moving toward a stationary state.

Using this information, we can argue that the majority of xanthan degradation and utilization occurs between the optical densities of 0.6 and 2.7, specifically at optical densities of 1.0 and 1.8. At the stationary phase, it is likely that the xanthan has been almost entirely utilized or degraded, explaining *Paenarthrobacter ilicis* strain 6C’s ability to reduce the culture medium viscosity to undetectable levels in the initial isolation experiments. The lack of mannose-6-phosphate isomerase class I (GMCHHGAN_003838) past an OD_600_ of 0.6 leads to it becoming undetectable by an OD_600_ greater than 2.7.

It is likely that although the culture supernatant can reduce the viscosity of a 1% xanthan solution to 40% of the initial value within 24 h (as seen in Fig. 2 in the viscosity section), the full degradation and utilization occur through a largely intracellular metabolic pathway and signal transduction that appears to push protein levels up and down depending on metabolite concentrations.

### Insights into the pPANIL 6C plasmid-derived xanthan utilization region and its significance

The recently characterized pPANIL 6C plasmid of *Paenarthrobacter ilicis* strain 6C harbors a unique xanthan utilization region that distinguishes this strain from previously known xanthan-degrading bacteria. This plasmid-borne XUR consists of approximately 17 genes and represents a specialized cluster encoding enzymatic, transport, and regulatory components for efficient xanthan metabolism. While exhibiting strong synteny and homology to the xanthan degradation genes present in *Microbacterium* sp*. XT11*, the pPANIL6C plasmid encodes a larger region with a genetically distinct putative xanthan lyase belonging to the PL8 family, underscoring the functional novelty of the plasmid pathway.

Although part of the plasmid is homologous to *Arthrobacter* sp*. FW305* unnamed plasmid CP084565, the XUR is unique and interestingly runs in an identical order to the *Microbacterium XT11* region (18) as shown in Fig. 8, but contains several genes not seen in the *Microbacterium XT11* xanthan degradation genomic region.

Notably, the XUR’s location adjacent to a mobF mobilization gene supports the hypothesis that this plasmid region is mobilizable, providing potential for horizontal gene transfer (36). This localization suggests an evolutionary advantage by potentially facilitating the dissemination of xanthan degradation capacity among diverse bacterial populations in environmental niches rich in xanthan. Specifically, the XUR exhibits a GC content (~59.9%) that is lower than both the chromosomal average (~62.8%) and the overall plasmid sequence (~61.4%). While the difference relative to the chromosome is statistically significant (permutation *P* = 0.027), the reduction relative to the plasmid falls within the expected variability of the plasmid’s composition (permutation *P* = 0.35). This pattern suggests that the XUR may represent a recently acquired genomic segment sourced from a donor organism with comparatively lower GC content (37). The shared presence of the adjacent mobF mobilization gene (GMCHHGAN_003835), a hallmark of conjugative transfer machinery, alongside a Type 4 secretion system conjugative DNA transfer family protein (GMCHHGAN_003867), further supports the hypothesis of horizontal gene transfer underlying the incorporation of this region. Together with syntenic conservation and proteomic evidence, these compositional data provide compelling evidence that the XUR is a distinct genomic entity embedded within an otherwise heterogeneous plasmid backbone. This finding underscores the mosaic and dynamic evolution of mobile genetic elements that confer adaptive metabolic capabilities.

Proteomic analyses of cultures grown on xanthan versus glucose reveal a well-orchestrated expression pattern among proteins encoded by the XUR. The putative xanthan lyase is expressed at low basal levels throughout most growth stages, detectable even at early optical densities (OD_600_ of 1.8), but it only accumulates to levels of significant abundance under xanthan conditions by mid-late exponential growth (OD_600_ of 1.8). This suggests a primed expression state that allows rapid induction in response to substrate availability rather than constitutive high-level expression, supporting efficient resource allocation.

Further evidence of inducible regulation is seen in the early appearance (OD_600_ of 1.0) of a lined cluster of ABC-type sugar transporters encoded by the XUR, which are essentially absent under glucose growth at all time periods. This pattern indicates sequential activation of substrate uptake systems followed by enzymatic degradation machinery, highlighting a finely tuned metabolic regulation likely responsive to extracellular xanthan or its degradation products.

Additional XUR-encoded proteins, including various glycoside hydrolases and multiple transcriptional regulators, demonstrate temporally distinct expression peaks and differential overabundance in xanthan cultures. The heterogeneity and temporal stagger of these expression profiles imply complex multilayered gene regulation adapting to dynamic substrate levels and metabolic demands during growth phases rather than a simple conditional operon.

Together, these data depict a recently evolved, highly regulated plasmid system conferring *Paenarthrobacter ilicis* strain 6C with a competitive edge in xanthan-rich environments, with potential for horizontal transfer due to the plasmid localization adjacent to mobilization genes. This discovery not only expands our understanding of plasmid-borne catabolic pathways but also highlights plasmids’ roles as vehicles for rapid adaptability and niche specialization in microbial communities.

The unique enzymatic capabilities, implied fine regulation, and potential mobility of the pPANIL6C plasmid-encoded XUR render it a promising target for biotechnological applications aimed at xanthan bioconversion, bioremediation, or industrial tailoring of polysaccharide properties.

### Proposing a putative model of *Paenarthrobacter ilicis* strain 6C xanthan degradation

Future experiments will be needed to elicit the specific qualitative activity of the enzymes involved to validate their position in the now putative xanthan degradation pathway. Additionally, transcriptomics experiments could be useful to better understand the specific induction and regulatory units within the XUR, but this goes beyond the scope of this paper.

**Fig 12:**
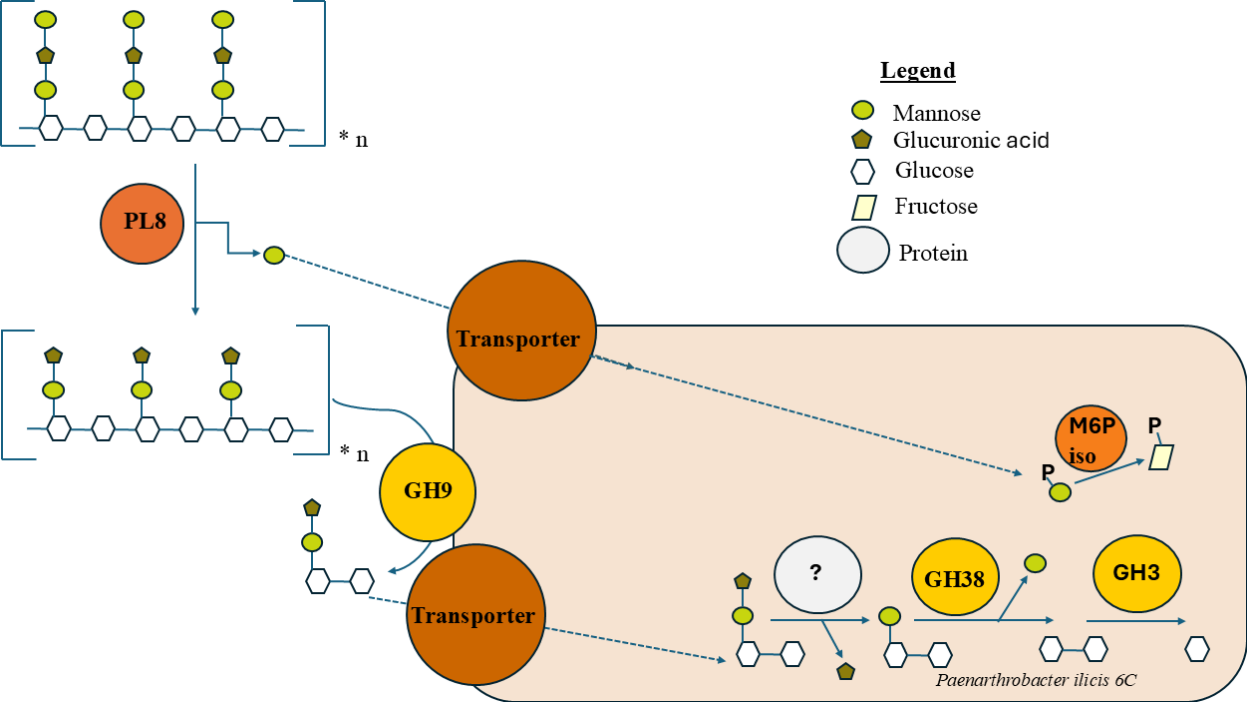
Visualization of the most likely putative xanthan degradation pathway seen in *Paenarthrobacter ilicis* strain 6C. PL8 is a xanthan lyase (GMCHHGAN_003836); the transporters are GMCHHGAN_003842, GMCHHGAN_003848, and GMCHHGAN_003849; GH9 is glycoside hydrolase family 9 protein (GMCHHGAN_003852); M6P iso is mannose-6-phosphate isomerase (GMCHHGAN_003838); GH38 is α-mannosidase glycoside hydrolase family 38 protein (GMCHHGAN_003845); GH3 is glycoside hydrolase family 3 protein (GMCHHGAN_003847), as shown with the EC number coloration established in Fig. 9. The legend defines the elements in the upper right hand side.

Based on the proteomics experiments and UniProt cellular localization prediction (transmembrane or secretion signal), the following putative xanthan degradation pathway has been proposed in Fig. 12. This pathway begins in the extracellular space where the secreted xanthan lyase PL8 (GMCHHGAN_003836) removes the terminal mannose on the xanthan side chain, which is likely directly imported via a phosphotransferase system. This mannose is likely to be imported based on the significant upregulation of the ABC Transporters (GMCHHGAN_003848 and GMCHHGAN_003849) and the mannose-6-phosphate isomerase (GMCHHGAN_003838) at the optical density of 0.6.

Following this, at an OD_600_ of 1.0, the now tetrameric repeating units of xanthan are likely cleaved from their repeating chains by the outer-membrane bound GH9 endoxanthanase (GMCHHGAN_003852), which becomes significantly overabundant by an OD_600_ of 1.8. Although there may be secondary activities seen by the xanthan lyase, it remains highly overabundant and significant at this growth point despite the loss of detectable mannose-6-phosphate isomerase class I. The resulting tetrameric or smaller fragments are then imported through some or all of the three transporters that are overabundant at the optical densities of 1.0 and 1.8.

Intracellularly, there has, at some point, been an activity to remove the glucuronic acid, although current structural predictions using bioinformatic methods cannot shed light on what enzyme is carrying out this potentially GH88 family protein type activity. Interestingly, the *Microbacterium XT11* also lacks a GH88 enzyme (18).

The oligosaccharide β-1,4 linked glucose residues are bound to a remaining mannose, with the mannose likely removed by the GH38 family protein (GMCHHGAN_003845), which is only significantly overabundant at the OD_600_ of 1.0. Finally, the remaining di-glucose backbone is broken by the GH3 family protein (GMCHHGAN_003847), which remains significantly overabundant through the experiment, even at an OD of 2.7. It seems likely that at an OD of 2.7, where the mannose-6-phosphate isomerase (GMCHHGAN_003838) and the N-acetyl glucosamine-kinase (GMCHHGAN_003837) are undetected, the initial steps are likely completed and much of the terminal mannose is already metabolized by an OD_600_ of 2.7.

### Conclusion

The newly discovered *Paenarthrobacter ilicis* strain 6C appears to conform to the knowledge base regarding *Paenarthrobacter* and overall comparability to *Paenarthrobacter A01* and *Paenarthrobacter nicotinovorans*, while increasing the number of potential bioactivities by adding xanthan degradation to the existing list. Additionally, *Paenarthrobacter ilicis* strain 6C appears to be the first member of the *Paenarthrobacter* genus, which has a flagella (GMCHHGAN_000805 to GMCHHGAN_000839) and is mobile.

Although the 4 Mb genome shares overall similarity to other *Paenarthrobacter* genomes, the 147 kB pPANiL_6C plasmid shares overall similarity to the *Arthrobacter* sp*. FW305* unnamed plasmid CP084565. However, the *Paenarthrobacter ilicis* strain 6C plasmid CP173686 has a unique region. This region is flanked downstream by a *mobF* mobilization protein, which contains genes coding for a novel xanthan utilization region. The genes within the XUR encode proteins that become almost entirely significantly overabundant at the optical densities of 1.0–1.8 when using xanthan compared to glucose as a sole carbon source.

This research establishes a novel *Paenarthrobacter ilicis* strain 6C pPANIL_6C plasmid that appears to contain a genetic region potentially sufficient to enable the use of xanthan as a sole carbon source. Additionally, it is very likely that this region contains either a novel enzymatic activity or a secondary activity by one of the enzymes to remove the glucuronic acid (GH88 family protein-type activity). However, this remains to be experimentally investigated and falls beyond the scope of the present study, similar to observations made in a proteomics analysis of *Microbacterium* XT11 (18).

This novel *Paenarthrobacter ilicis* strain 6C offers the ability to expand the compendium of xanthan-degrading microorganisms and offers the genomic and amino acid coding for a handful of new enzyme configurations that can be further quantified and modified. Additionally, these findings expand the knowledge base regarding the *Paenarthrobacter* genus and its capabilities.

## MATERIALS AND METHODS

### Strain isolation method and media composition

The search for the identification of novel xanthan degraders and degrading enzymes was adapted from the methods of references 17 and 19. As opposed to the established methods, the chosen media utilized xanthan as a sole carbon source with the aim of identifying fewer false positives and increasing the probability of finding genera missed in prior studies.

The chosen medium (M9X) consisted of M9 minimal medium with values given per liter, with effective concentration given in parentheses. Unless otherwise mentioned, all reagents for the medium were obtained from Merck with values given in the stock solution and (media concentration): 100 mL M9 salt solution (10×) (Na_2_HPO_4_ 33.7 mM [0.337 mM], KH_2_PO_4_ 22.0 mM [0.22 mM], NaCl 8.55 mM [0.00855 mM], and NH_4_Cl 9.35 mM [0.0935 mM]), cell-free xanthan 0.5% (wt/vol) (Jung-Bunzlauer AG), 1 mL of 1 M MgSO_4_ (1 mM), 0.3 mL of 1 M CaCl_2_ (0.3 mM), 1 mL of biotin (1 mg/mL) (1 µg/mL), 1 mL thiamin (1 mg/mL) (1 µg/mL), 10 mL of trace elements solution (100×) (containing EDTA trisodium monohydrate 5 g /L = 13.4 mM [0.134 mM], FeCl_3_·6H_2_O 0.83 g/L = 3.1 mM [0.031 mM], ZnCl_2_ 84 mg/L = 0.62 mM [0.0062 mM], CuCl_2_·2H_2_O 13 mg/L = 76 µM (0.00000076 mM), CoCl_2_·2H_2_O 10 mg/L = 42 µM (0.00000042 mM), H_3_BO_3_ 10 mg/L = 162 µM (0.00000162 mM), and MnCl_2_·4H_2_O 1.6 mg/L = 8.1 µM (0.000000081 mM).

LB medium for single colony restreaking was composed of 10 g/L tryptone (Sigma-Aldrich), 5 g/L yeast extract (Sigma-Aldrich), and 5 g/L NaCl (VWR).

**Fig 13:**
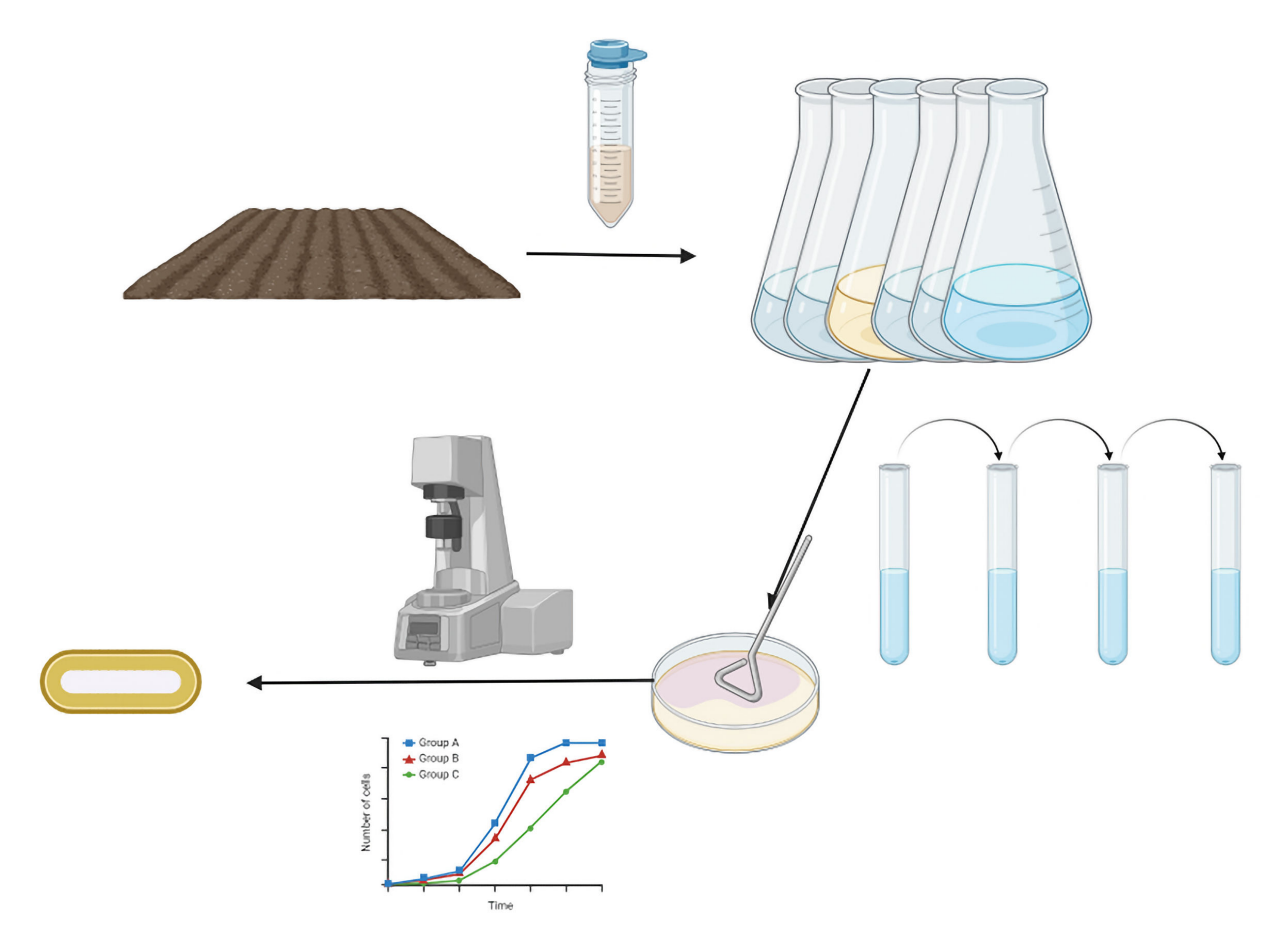
The sampling and selection process workflow, which was based on viscosity as measured in a rheometer and growth as measured via the optical density at 600 nm. Dilution from the most “successful” flask was used to identify promising single colonies that were evaluated as described.

Soil samples were obtained from the surface soil of a private garden in Bielefeld, NRW, Germany, and taken to Bielefeld University for processing. Soil samples were diluted in 2.5× (wt/vol) of PBS at pH 7.0, rolled on a roller for 30 minutes for homogenization, then centrifuged at 500 × *g* for 5 minutes, with 1 mL of the resulting supernatant used to inoculate six flasks containing 50 mL of M9X medium. These flasks were incubated at 28°C with 140 rpm for 3 days and then evaluated via testing of the optical density at 600 nm and the change in viscosity versus sterile M9X media. The best-performing flask (high OD_600_ and lowest relative viscosity) was chosen for additional isolation steps and basic characterization. The general workflow for the enrichment and isolation is shown in Fig. 13.

### Determination of viscosity changes using sterile supernatant in an *in silico* xanthan solution environment

The identification of biologically relevant novel xanthan degraders was based primarily on the growth of the isolates in combination with the change in viscosity of the culture medium. Viscosity was measured in millipascals using a Rheotec Viscometer rotating once per minute. Samples were compared to sterile M9X culture medium, and results are provided as a relative value.

Viscosity testing using sterile supernatant of the isolated strain was undertaken with 10 mL of 1% xanthan containing 1× M9 salts, with 200 µL of sterile filtered culture supernatant added and then measured once an hour for 24 h while being continuously stirred in a Rheotec Viscometer at one rotation per minute using a TL7 head at 34°C. This experiment was followed by a control run with *E. coli*, which failed to change the viscosity.

### Cultivation of *Paenarthrobacter ilicis* strain 6C

*Paenarthrobacter ilicis* strain 6C was cultivated in M9 medium containing cell-free xanthan obtained from Jungs-Bunzlauer AG, diluted to 0.3% (wt/vol). Cultures were inoculated at an optical density of 0.1 at 600 nm from M9 minimal media with the relevant carbon source. The inoculum was prepared from cryoculture 24 h in advance for glucose media and 48 h in advance for xanthan media, then diluted and pipetted into flasks or 48-well flower plates. Cultures in flasks were used as reference points with which the biolector data were corrected (linearity = 0.99), and optical density was measured in plastic cuvettes at 600 nm in a spectrophotometer, and dilutions were made with 1× M9 Salts in distilled water or 1× PBS.

Precultures of inoculum of the subsequent carbon source were inoculated at an OD_600_ of 0.1 for the main cultures used for optical density and proteomics measures.

### Measuring motility

Motility was determined using 0.35% agar LB plates in 24 h time periods for 72 h, whereby approximately 1 µL of preculture (as described in Cultivation of *Paenarthrobacter ilicis* strain 6C) was applied to the center of the plate. Growth was compared with *Xcc B100-FliC* as a negative control (38).

### Gram staining

Gram staining was performed using a Gram staining kit and the method as prescribed by Carl Roth GmbH, Germany.

### Physiological optima determination

In order to determine the physiological optima (temperature and pH), *Paenarthrobacter ilicis* strain 6C was cultivated in triplicate flasks for each condition. Each experimental condition was compared with the initial cultivation conditions (30°C and pH 7.0) to ensure replicability based on the comparability of the control/initial conditions. The pH of the M9 minimal media was adjusted using 5 M HCl (hydrochloric acid) and 5 M NaOH (sodium hydroxide). Triplicate flasks were used for each discrete step in pH, as well as for each 5°C increment in temperature.

The growth rate *μ* was calculated as the natural logarithm (ln) difference in the optical density over a specific time:


μ=[ln(n1)−ln(n0)]/ t.

### Enteropluri test

The EnteroPluri test was applied as described by Liofilchem Diagnostic using duplicates, each with a single colony. This assay aims to provide a basic physiological characterization of glucose fermentation, lysine decarboxylation, ornithine decarboxylation, adonitol fermentation, lactose fermentation, arabinose fermentation, sorbitol fermentation, acetoin production, dulcitol fermentation/phenylalanine deamination, urea hydrolysis, and citrate utilization.

### Catalase test

Catalase activity was tested using cells from a plate streaked on a microscope slide and treated with a 3% H_2_O_2_ solution. *E. coli K12* was used as a positive control, and only H_2_O_2_ was used as a negative control.

### Figure creation software

The genome overlap figure of pPANIL6C with CP084565 (Fig. 7) was generated with a custom Python workflow using Biopython (SeqIO) (39), gffutils (gffutils: a Python package for working with and manipulating the GFF and GTF format files. PyPI, https://pypi.org/project/gffutils/), dna_features_viewer (CircularGraphicRecord/GraphicFeature) (40), and Matplotlib (41); GC/skew was computed in sliding windows, and BLASTn (42) was called from the script to render identity bands and labels. The code for the plasmid overlap script can be found at https://github.com/michaelcthomas2000-debug/Plasmid-commonality-and-ROI-mapper/blob/main/V1pt1.

The regional overlap figure with MXT11 (Fig. 8) was created using pygenomeviewer (pygenomics) (22) together with NCBI BLAST (42).

### Sporulation and anabiosis (metabolic hibernation)

To determine sporulation, cultures were allowed to grow under nitrogen limitation (left at the stationary phase of OD 4.0 for 72 h) and allowed to dry out completely before being stained with the Schaffer-Fulton method of endospore staining. A culture of *Bacillus megaterium* was also raised under nitrogen limitation as a positive control, and *E. coli* was grown as a negative control.

Desiccation experiments were carried out to provide a precursory exploration of environmental persistence and anabiosis, which was undertaken using limited culture media in flasks that were allowed to completely evaporate and then shaken further for 3 days. These flasks were then given fresh media, incubated for a day, and then the media were removed and applied to M9 xanthan agar plates to measure the capability to survive desiccation and starvation.

### Anaerobic growth

A 3.5 L Oxoid anaerobic chamber (Oxoid Deutschland GmbH) was used with an Aerocult C strip (Merck). Additionally, the chamber was flushed 12 times using N_2_ gas to create a maximally oxygen-free environment. Plates were inoculated by streaking from cultures grown at 30°C for 2 days, and *E. coli K12* was used as a positive control, known as a facultative anaerobe. Triplicate plates were incubated at 30°C for 5 days, and growth was evaluated visually; photographs can be found in the supplemental material.

### Scanning electron microscopy

Scanning electron microscopy was undertaken using a Hitachi S-450 scanning electron microscope (Hitachi, Japan), whereby samples were first prepared on a Poretics (0.2 µm polycarbonate membrane) filter membrane and then imaged at 10,000×.

Bacteria from the late exponential growth phase were harvested on Poretics (0.2 µm polycarbonate membrane) filters. The cells were fixed using 2% glutaraldehyde (Sigma-Aldrich) in phosphate buffer (100 mM and pH 7.2) for 10 minutes. Filters with bacteria were air dried and sputtered with 10 nm gold using an SCD005 sputter (BalTec, Wetter, Germany). The samples were analyzed using a Hitachi S-450 scanning electron microscope at 15 kV. Images were recorded using a Pointelectronics (Halle, Germany) digitizer.

### DNA isolation

Initial DNA of a yellow colony from a cabbage patch provided DNA for 16s sequencing, which was obtained via the Universal DNA isolation method (43) and LPW57 and LPW58 primers (44). Genomic DNA of strain *P. ilicis 6C* was isolated using the NucleoSpin Microbial DNA Kit (MACHEREY-NAGEL GmbH & Co. KG) as prescribed by the user manual for gram-positive bacteria.

### DNA sequencing

Initial identification was undertaken using LPW57 and LPT58 primers for the 16s-23s ribosomal region, and sequencing was done using the Sanger method by the Bielefeld Center for Biotechnology and Blastn.

Long DNA reads were generated by Nanopore sequencing. For library preparation, the SQK-LSK112 sequencing kit (Oxford Nanopore Technologies) was used without prior shearing of the DNA. Sequencing was performed on a GridION platform using a R10.4 flow cell. Base calling and demultiplexing were performed using GUPPY version 6.4.2 with the super-accurate basecalling model version 2021-11-17. Reads were then trimmed with CUTADAPT (45) with parameters -e 0.2 --trimmed-only and -g AAGGTTAANNNNNNNNNNNNNNNNNNNNNNNNCAGCACCT, followed by a second invocation of CUTADAPT with parameters -e 0.2, -m 1000, and -a AGGTGCTGNNNNNNNNNNNNNNNNNNNNNNNNTTAACCTTAGCAAT.

From the trimmed reads, the assembly was generated using FLYE version 2.9 (46). The resulting two contigs representing the circular genome and the circular plasmid were annotated using the PGAP pipeline (47, 48).

### Gene enrichment analysis

For each time point, we built condition-specific matrices (glucose versus xanthan) from the pellet fraction; for two pre-specified ROI proteins predicted to be secreted, namely the GH9 and xanthan lyase PL8 proteins (GMCHHGAN_003836 and GMCHHGAN_003852), we substituted Supernatant LFQ values when a 1:1 replicate structure was available. Protein identifiers were extracted from MaxQuant identifier fields by regex (GMCHHGAN_\d{6}), and a region-of-interest comprising 16 adjacent genes (GMCHHGAN_003836–GMCHHGAN_003852) was defined *a priori*.

Statistics were performed per protein and time point. Raw intensities were first used to impute a missing third replicate by the mean of the other two (row-wise; applied only when exactly two values were present, as for the proteomics data). Data were then transformed to log2 (zeros → NaN). When enabled, missing log2 values were down-shift imputed to (column minimum − 1.0) before averaging replicates. A protein was considered valid for testing if it had ≥2 non-missing replicates in at least one condition. Log2 fold change (default reported as xanthan − glucose; orientation configurable) was computed from replicate means. To test ROI enrichment, we compared the distribution of log2FC values for ROI proteins versus all other valid proteins at each time point using both Welch’s *t*-test and the Kolmogorov–Smirnov two-sample test; we report group sizes, means, and test *P*-values.

### Regional GC composition of the XUR region

We tested whether the GC content of a predefined plasmid locus deviates from (i) the GC content of the plasmid that harbors it and (ii) the organism-wide GC content (chromosome + plasmid). Analyses were performed with a custom Python script (NumPy [49]; SciPy [50] [exact binomial test when available]) that reads a plasmid FASTA (single sequence) and a genome FASTA (all contigs concatenated).

The ROI was defined on plasmid coordinates either directly by 1-based inclusive coordinates or programmatically from feature annotations. For GFF input, we supported (i) collapsing all features that share a given attribute (e.g., ID = XUR) to their min-max span and (ii) defining a span by a start feature and an end feature (e.g., locus_tag = xurA → locus_tag = xurZ), restricted to a specified seqid. For the XUR locus analyzed here, the ROI covered positions 90,941–116,766 on plasmid pPANIL_6C (no wrap-around); we treated the plasmid as circular for random-window sampling.

All calculations exclude ambiguous bases (i.e., only A/T/G/C are considered “valid”). For any sequence S, GC proportion is *p* = #(G,C)/#(A,T,G,C). Two baselines were used (i) the plasmid-wide GC proportion computed from the plasmid FASTA, and (ii) the genome-wide GC proportion computed from the concatenated genome FASTA (chromosome plus plasmid sequences for *Paenarthrobacter ilicis* strain 6C). Effect sizes are reported as absolute differences Δ*p* = *p*_ROI − *p*_baseline (percentage-point interpretation).

For each baseline, we performed two complementary, two-sided tests:

1. Binomial test. Under *H*0: *p* = *p*0 (with *p*0 ∈ {plasmid GC, genome GC}), the number of GC bases in the ROI (*k*) follows Binomial(*n*, *p*0) with *n* = # valid bases in the ROI. We report the exact two-sided *P*-value (SciPy’s binomtest) or a continuity-corrected normal approximation when SciPy is unavailable. A 95% Wilson score interval is provided for p_ROI.
2. Permutation (empirical) test. To relax independence assumptions and account for composition heterogeneity, we drew *N* = 200,000 random windows from the same background sequence as the null (plasmid or whole genome). Windows were selected uniformly with span lengths chosen so that the number of valid bases was at least the ROI’s valid length. For each window with GC proportion p_i, we used the two-sided extremeness criterion |*p*_*i* − *p*0| ≥ |*p*_ROI − *p*0| and computed the add-one smoothed empirical *p*-value: *p*_perm = (1 + #extreme)/(*N* + 1).

Plasmids were treated as circular for sampling; the genome baseline used the concatenated sequence, which is appropriate under a stationary-composition null. *N* = 200,000 permutations; random seed = 47. The script writes a tab-separated table with per region and per baseline: ROI GC, baseline GC, Δ*p*, Wilson 95% CI, binomial *p*, BH-FDR *q* (if multiple regions), and the empirical permutation *p*. All inputs (FASTA paths, region definitions, and toggles) are specified in a user-configurable header in the script to ensure exact reproducibility.

### Proteome analysis

Samples were processed to provide two fractions: namely, the sterile supernatant fraction and the cell pellet fraction. Samples were taken at the given optical densities and centrifuged at 8,000 × *g* for 5 minutes, providing cell pellets that were washed with comPLETE Protease Inhibitor (Roche) in PBS, centrifuged again at 8,000 × *g* for 5 minutes, and then frozen in liquid nitrogen to be stored at −80°C before lyophilization. The supernatant was filtered through a sterile 0.2 µm filter (FisherSci) to obtain the extracellular proteins and then frozen at −80°C prior to lyophilization.

Approximately 6–12 mg of freeze-dried material was then weighed out for each sample, with pellets being lysed via mechanical destruction in 1 mL of 100 mM AmBic (ammonium bicarbonate from Sigma) in a Ribolyzer (Bertin Precellys 24) with 0.1 mm beads (BioSpec Products) at 6,500 rpm for 30 seconds with three repeats, and samples were cooled on ice between each of the three phases. Following the lysis, pellet samples were then frozen in liquid nitrogen and freeze-dried. All samples were then processed with 100 µL of trifluoroethanol (TFE, VWR Chemicals), 100 µL of AmBic with 200 µM dithiothreitol (DTT, VWR Chemicals), and 200 µM of Iodoacetamide (IAA, Sigma). Specifically, 5 µL of the DTT solution was added to the samples, they were inverted, and then incubated for 60 minutes at 60°C. Next, there was an addition of 20 µL of the IAA solution prior to inversion and incubation for 90 minutes in the dark at RT. Finally, an additional 5 µL of the DTT solution was added to the samples, inverted, and then incubated for another 60 minutes at RT.

The resulting TFE-treated samples were then prepared for tryptic digestion, whereby 437 µL of AmBic and 437 µL of distilled water were added to each sample in addition to 6 µL (1 µg/µL) of trypsin (Trypsin Gold, Promega) before incubation at 37°C overnight, resulting in digested peptide samples.

The peptide samples were then cleaned using the filter-assisted sample preparation method (51) using a Sep-Pak C18 1 cc Vac Cartridge from Waters Conglomerate (WAT054955), dried in a ThermoFisher Speed-Vac, and then redissolved in 98% MS-water with 2% acetonitrile and 0.1% trifluoroacetic acid (VWR Chemicals). The samples were then diluted between 0.5 and 1.5 µg/µL, and the mass spectra were obtained via ThermoFisher Q-Exactive plus LC-ESI-MS with a linear gradient going from 3.2% to 76% acetonitrile (relative to water) with 0.1% formic acid over 80 minutes. The Orbitrap was run in positive mode from 5 to 92 minutes at a resolution of 70,000 and an AGC (advanced gain control) of 3e6 ions, with a maximum injecting time (IT) of 64 ms and a scan range of 350–2,000 *m/z*. Data were generated using a data-dependent method, selecting the top 10 peaks at a maximum IT of 100 ms, an AGC of 2e5, an isolation window of 1.6 *m/z,* and a resolution of 17,500. The sample AiGS32 (second replicate for the third time point for glucose supernatant) was run twice, and the 4_29 marker was chosen for further processing instead of the 2_29 version of the file, and only the 4_29 version was included in the statistical evaluation.

The resulting .raw files were analyzed in MaxQuant version 2.6.6 with a protein defined as identified if two unique peptide fragments are identified and it is seen in at least two samples, with quantification using LFQ via peptide correlations. Other than matching between runs, default settings were used. The resulting label-free quantification was transformed via log(2), non-valid values were replaced with 1, and missing triplicate values were imputed from the other two existing values as the mean. The data were analyzed using SciPy and Matplotlib in Python and visualized with the help of volcano plots (using Welch’s *t*-test) using an FDR of 0.05.

QC proteins were identified for experimental biases by checking by “Name Convention”: genes that code for ribosomal proteins almost always start with specific prefixes: rpl (for the Large subunit), rps (for the Small subunit), or rpm (for Mitochondrial ribosomal proteins). If a gene name starts with one of these, it is flagged as ribosomal. This is a very reliable, rule-based method.

QC proteins were also identified by checking by Description: as a backup and to catch any proteins that might not follow the naming rule perfectly, the protein’s full description (the “product” field) was scanned. If a keyword like “ribosomal” or the terms “30S” or “50S” occurs (which refer to the two main parts of a bacterial ribosome), they are also flagged as ribosomal proteins.

We are confident in this method because it combines a strict, standardized naming system with a broader keyword search of the description, making it very unlikely to either miss a true ribosomal protein or incorrectly label something else.

For chaperones, the most common and well-known chaperone protein names, such as GroEL, DnaK, ClpB, GrpE, and others, were marked. These are famous “heat shock proteins” that help other proteins fold correctly.

The gene name and protein description for every protein in the data set were checked.

We are sure this works because these specific chaperone names are highly conserved across many species and are staples of molecular biology. A protein identified as “DnaK” in a bacterial proteomics data set is almost universally the DnaK heat shock chaperone. This method is direct and relies on identifying proteins by their universally recognized names.

In short, we identify these QC proteins by recognizing standard, text-based identifiers, not by guessing. It is a classification based entirely on established scientific nomenclature.

### Statistical evaluation

Statistical evaluation was performed using Welch’s *t*-tests with the assumption of unequal variance (heteroskedastic) because some proteins showed 0 variance (undetected) and needed to be compared to replicate groups with high values and comparatively high variance.

Unless a SignalP 6.0 (52) analysis determined a secretion signal, intracellular localization was assumed, and only these values were used in statistical analysis in the event of intracellular proteins winding up in low abundance in the supernatant samples.

For the *t*-tests, in the event a single biological replicate had 0, but the other two replicates had valid values, then the mean was imputed to replace the 0. For the Log2 used in the *t*-tests, values were calculated with 0s replaced using the minimal measured Log2 value within the entire data set and then subtracting one. Ratios between conditions were calculated using the original LFQ intensity values without any values being imputed or changed.

The gene enrichment analysis was undertaken using the MaxQuant .txt file, whereby for each time point, it uses the same hybrid data as the proteomics analysis (from pellet values taken unless identified as secreted). The proteins in the ROI and “Rest of Genome” groups were compared and used in statistical tests (*t*-test and K-S test) to determine if the fold change behavior of the ROI group is significantly different from that of all other proteins.

## Data Availability

The trimmed genomic data are available from SRA via BioProject PRJNA1182270. The annotated chromosome and plasmid are available under the GenBank accession numbers CP173685 and CP173686. The mass spectrometry proteomics data have been deposited to the ProteomeXchange Consortium via the PRIDE partner repository with the dataset identifier PXD063987 and https://dx.doi.org/10.6019/PXD063987 (53).

## SUPPLEMENTAL MATERIAL

Supplemental Document spectrum.01690-25-s0001.docx Figures S1 to S4 and Tables S1 to S4.

Supplemental Data Sheets spectrum.01690-25-s0002.xlsx Data Sheets S1 to S7.

ASM does not own the copyrights to Supplemental Material that may be linked to, or accessed through, an article. The authors have granted ASM a non-exclusive, world-wide license to publish the Supplemental Material files. Please contact the corresponding author directly for reuse.
