# Erratum for Thomas et al., “Genomic and proteomic characterization of a newly isolated *Paenarthrobacter ilicis* strain and its plasmid-mediated xanthan degradation”

**DOI:** 10.1128/spectrum.00075-26

**Published:** 2026-03-20

**Authors:** Michael Thomas, Andreas Schlüter, Jenny Fjodorova, Christian Rückert, Tobias Busche, Karsten Niehaus

## ERRATUM

Volume 14, no. 1, e01690-25, 2026, https://doi.org/10.1128/spectrum.01690-25. Figure 3 caption: “…panel A showing growth for 24 h to highlight growth in glucose M9 media and panel B showing the full 96 h to highlight the growth in xanthan M9 media” should read “…panel A showing the full 96 h of growth and panel B showing the first 24 h of growth to highlight the faster growth in glucose M9 media.”

Table 2, row 5: “85.98” should appear in the blank space under “AA% identity.”

Materials and Methods, "Determination of viscosity changes using sterile supernatant in an *in silico* xanthan solution environment": “…supernatant in an *in silico...*” should read “…supernatant in a stirring…”

These changes did not affect any conclusions within the paper.

