# Supplementary material for: Genomic and proteomic characterization of a newly isolated Paenarthrobacter ilicis strain and its plasmid-mediated xanthan degradation
Source: Microbiol Spectr. 2025 Dec 10;14(1):e01690-25. doi: 10.1128/spectrum.01690-25 (PMC12772297; doi:10.1128/spectrum.01690-25)
Supplement: Supplemental Document — Figures S1 to S4 and Tables S1 to S4. [file spectrum.01690-25-s0001.docx]

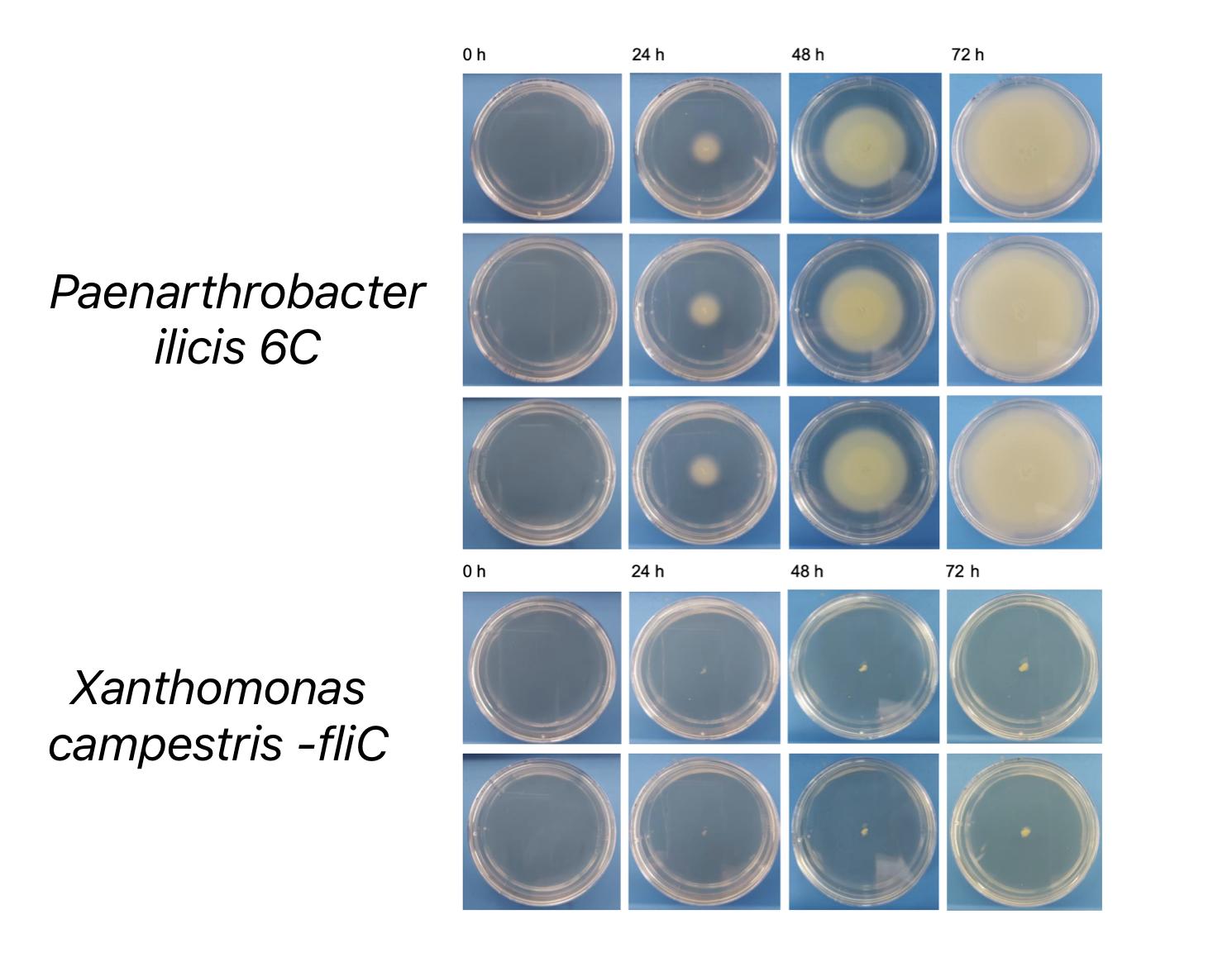


Supplemental Figure 1: Motility assay using 0.3% agar LB plates left at 30° C for 72 h. *Xcc -Flic* (20) was used as a negative motility control.


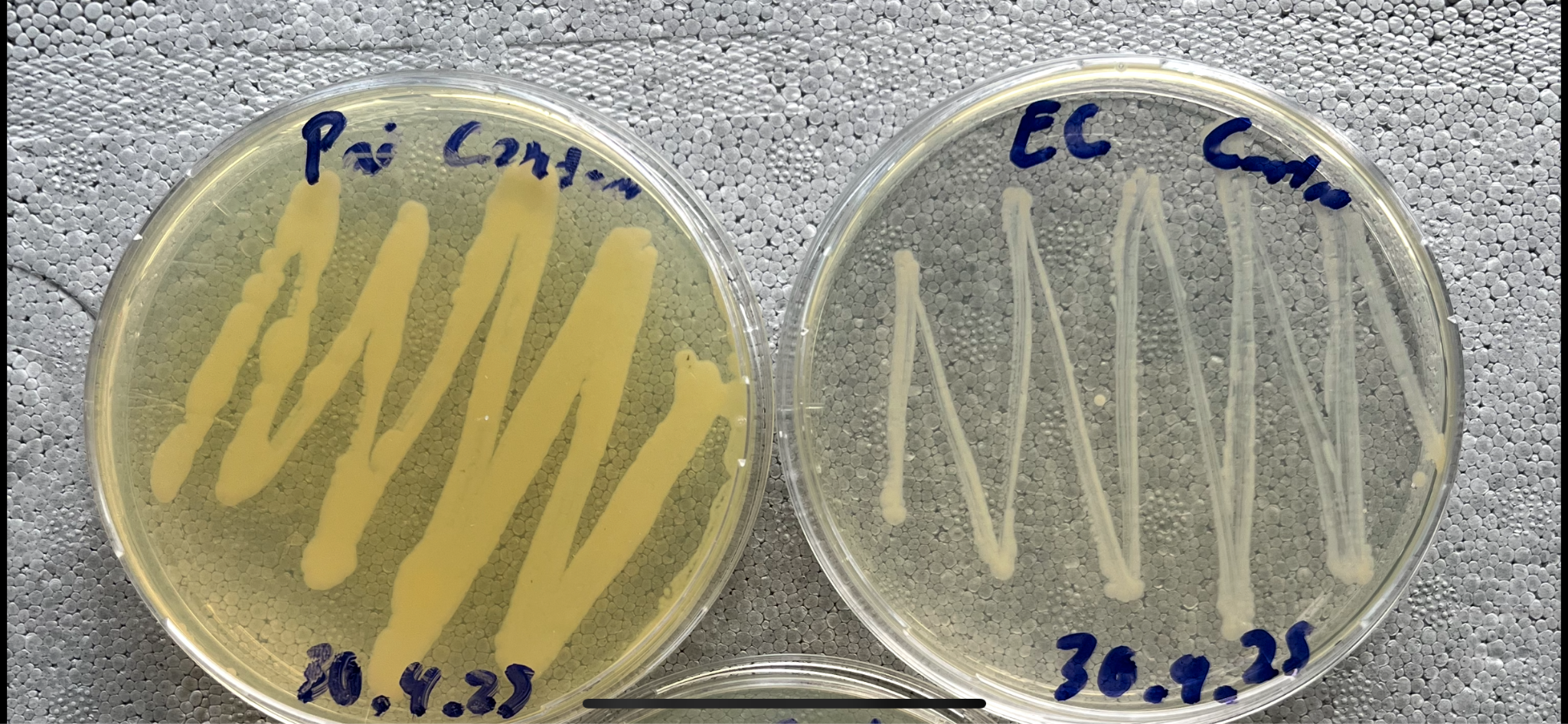


Supplemental Figure 2: Exemplary aerobic positive control for *Paenarthrobacter ilicis* 6C and *E. coli* K12 to show viability of plates, streaking form, and strains (16 h).


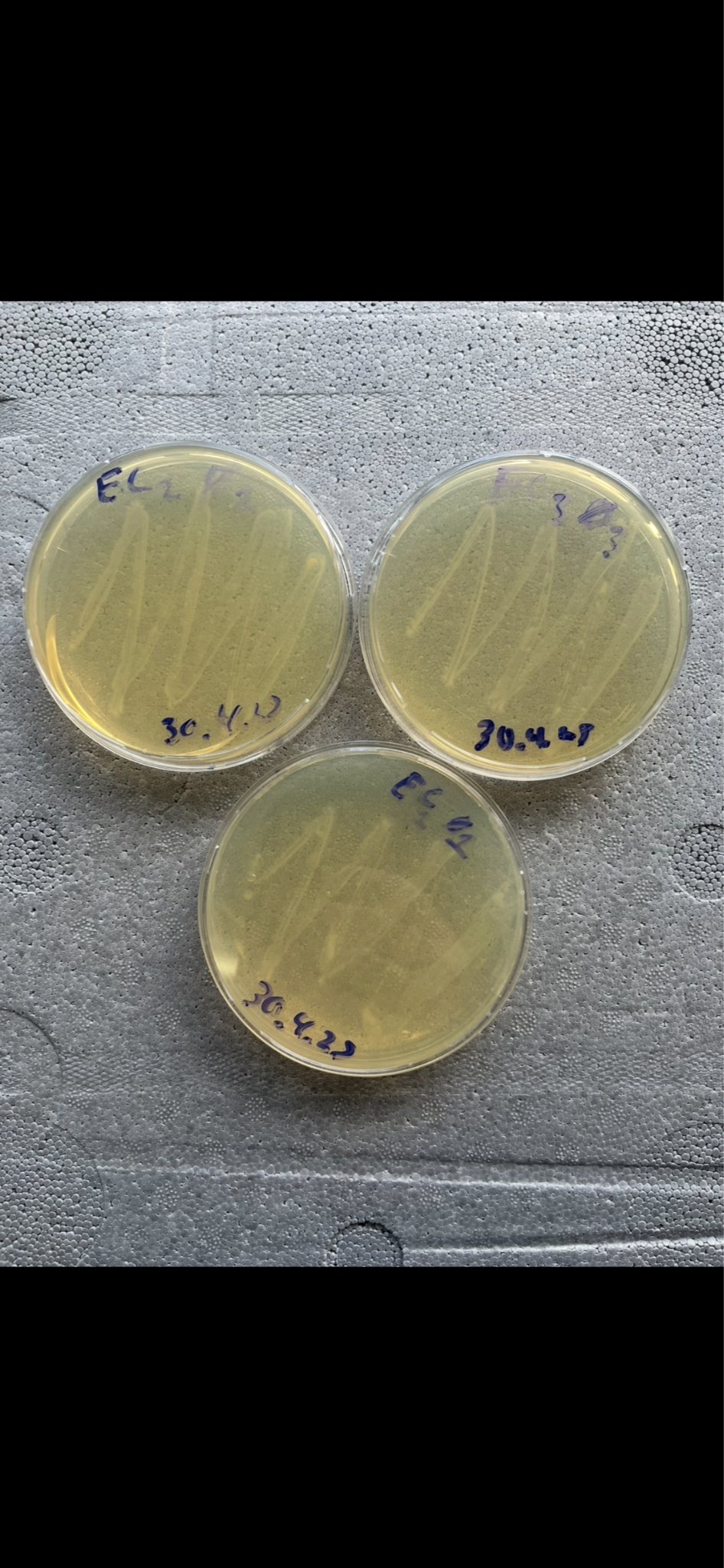


Supplemental Figure 3: *E. coli* K12 incubated at 30° without oxygen for 120 h. Triplicates streaked from the same plate as the control (Supplemental Figure 2)#


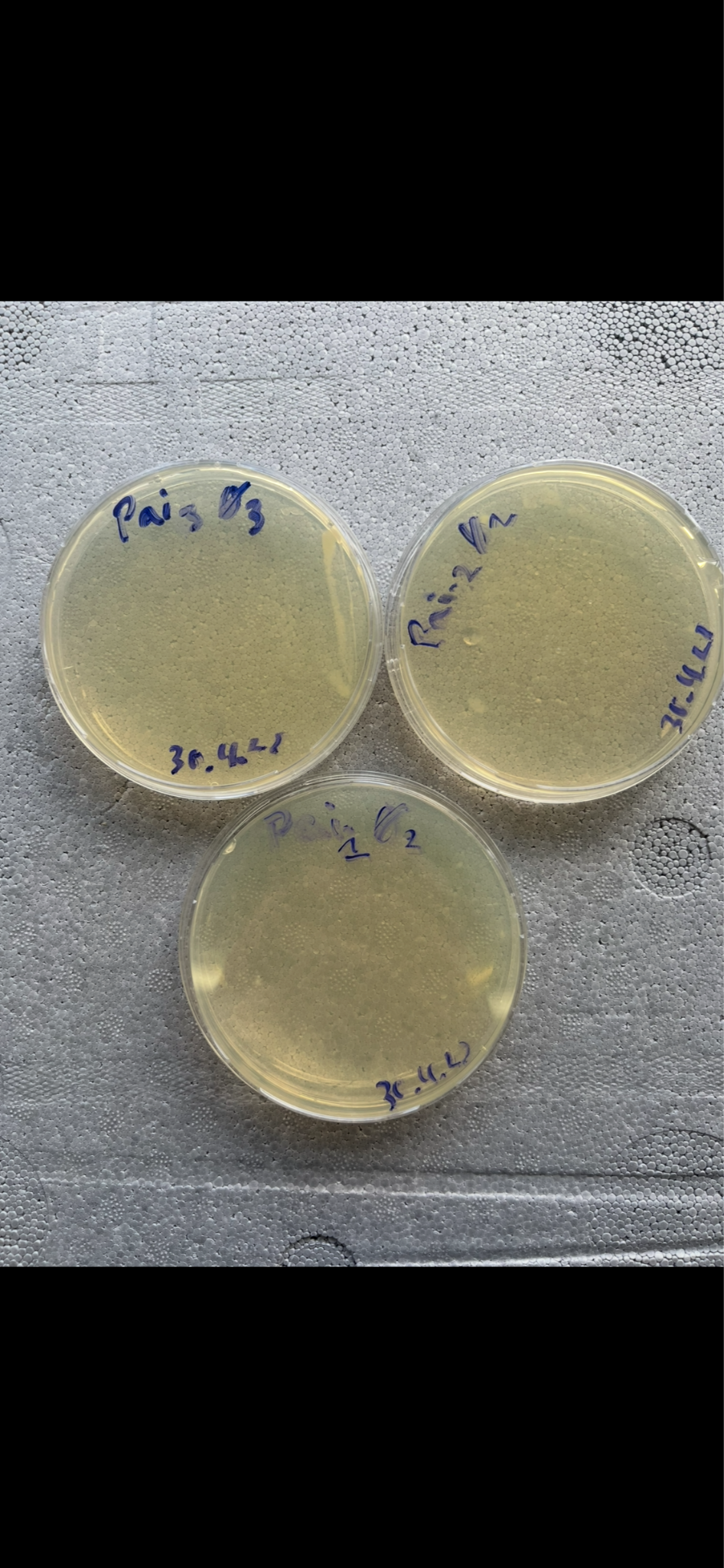


Supplemental Figure 4: *Paenarthrobacter ilicis* 6C incubated at 30° without oxygen for 120 h. Triplicates streaked from the same plate as the control (Figure 2).

Supplemental Data:
To show this data in another way, significant proteins will be presented in tables and insignificant or undetected proteins will be listed afterwards. For the comparison of protein abundance differences at an OD_600_ of 0.6, 53% of the proteins in the XUR were significantly overabundant (8/15) (Supplemental Table 1), ordered by their annotated functional group and predicted activity.

Supplemental Table1: Proteins encoded within the XUR located on the 147kb pPANIL_6C plasmid which are significantly overabundant in the xanthan condition at an OD_600_ of 0.6

| Protein Name | Accession | Fold Change / Detection | P-Value |
| --- | --- | --- | --- |
| Glycoside hydrolase family 9 protein | GMCHHGAN_003852 | Overabundant by 42.90x | 0.007 |
| Glycoside hydrolase family 3 protein | GMCHHGAN_003847 | Overabundant by 21.44x | 0.017 |
| Mannose-6-phosphate isomerase, class I | GMCHHGAN_003838 | Only in xanthan condition | 0.00024 |
| N-acetylglucosamine kinase | GMCHHGAN_003837 | Only in xanthan condition | 0.00058 |
| ABC transporter solute-binding protein | GMCHHGAN_003842 | Overabundant by 22.82x | 0.006 |
| Extracellular solute-binding protein | GMCHHGAN_003850 | Overabundant by 9.44x | 0.02 |
| FAD-dependent oxidoreductase | GMCHHGAN_003843 | Overabundant by 42.37x | 0.0017 |
| LacI family DNA-binding transcriptional regulator | GMCHHGAN_003844 | Only in xanthan condition | 0.0000373 |

The rest of the proteins encoded within the XUR were non-significantly overabundant in the xanthan condition, namely the polysaccharide lyase 8 family protein (GMCHHGAN_003836) was detected. The substrate-binding domain-containing protein (transcription factor) (GMCHHGAN_003853), α-mannosidase GH38 (GMCHHGAN_003845), and the LacI family DNA-binding transcriptional regulator (GMCHHGAN_003839) were also detected. Meanwhile, the carbohydrate ABC transporter permease (GMCHHGAN_003840), carbohydrate ABC transporter permeases (GMCHHGAN_003848 and GMCHHGAN_003849), and hydroxyacid dehydrogenase (GMCHHGAN_003851) were not detected.

At an OD_600_ of 1.0, 13 out of 15 (80%) of the proteins detected at any sampling time point in the XUR were significantly overabundant (Supplemental Table 2). All proteins detected at this growth point encoded within the XUR were found to be significantly overabundant in the xanthan condition.

Supplemental Table 2: Proteins encoded within the XUR located on the 147kb pPANIL_6C plasmid which are significantly overabundant in the xanthan condition at an OD_600_ of 1.0

| Significant Proteins at OD600 of 1.0 |  |  |  |
| --- | --- | --- | --- |
| **Protein Name** | **Accession** | **Fold Change / Detection** | **p-Value** |
| Polysaccharide lyase 8 family protein | GMCHHGAN_003836 | Overabundant by 200.07× | 0.031 |
| Glycoside hydrolase family 9 protein | GMCHHGAN_003852 | Overabundant by 6236.74x | 0.01739 |
| Glycoside hydrolase family 3 protein | GMCHHGAN_003847 | Overabundant by 275.04× | 0.0185 |
| α-mannosidase GH38 | GMCHHGAN_003845 | Overabundant by 3905.02× | 0.00138 |
| ABC transporter solute-binding protein | GMCHHGAN_003842 | Overabundant by 32.91× | 0.00058 |
| Carbohydrate ABC transporter permease | GMCHHGAN_003848 | Only in xanthan condition | 0.000171 |
| Carbohydrate ABC transporter permease | GMCHHGAN_003849 | Only in xanthan condition | 0.0000821 |
| Extracellular solute-binding protein | GMCHHGAN_003850 | Overabundant by 77.31× | 0.00221 |
| Substrate-binding domain-containing protein (transcription factor) | GMCHHGAN_003853 | Overabundant by 9.35× | 0.000173 |
| FAD-dependent oxidoreductase | GMCHHGAN_003843 | Overabundant by 59.76× | 0.00275 |
| Hydroxyacid dehydrogenase | GMCHHGAN_003851 | Only in xanthan condition | 0.0000499 |
| LacI family DNA-binding transcriptional regulator | GMCHHGAN_003839 | Overabundant by 1.88× | 0.000245 |
| N-acetylglucosamine kinase | GMCHHGAN_003837 | Only in xanthan condition | 0.0000608 |

The remaining 3 out of 15 proteins detected at any point within the XUR were undetected at this time point, namely the mannose-6-phosphate isomerase, class I (GMCHHGAN_003838) and the LacI family DNA-binding transcriptional regulator (GMCHHGAN_003844).

When the cells had reached an optical density at 600nm of approximately 1.8, 11 of the 15 proteins (66.6%) detected at any point within the XUR were significantly overabundant in the xanthan condition, and all but one of the proteins encoded within the XUR detected at this growth phase were significantly overabundant in the xanthan condition (Supplemental Table 3

Supplemental Table 3: Proteins encoded within the XUR located on the 147kb pPANIL_6C plasmid which are significantly overabundant in the xanthan condition at an OD_600_ of 1.8

| Protein Name | Accession | Fold Change / Detection | p-Value |
| --- | --- | --- | --- |
| Polysaccharide lyase 8 family protein | GMCHHGAN_003836 | Overabundant by 786.70× | 0.00558 |
| Glycoside hydrolase family 3 protein | GMCHHGAN_003847 | Overabundant by 2481.45× | 0.00339 |
| Glycoside hydrolase family 9 protein | GMCHHGAN_003852 | Only in xanthan condition | 0.000171 |
| Carbohydrate ABC transporter permease | GMCHHGAN_003848 | Only in xanthan condition | 0.0000451 |
| Carbohydrate ABC transporter permease | GMCHHGAN_003849 | Only in xanthan condition | 0.0000547 |
| Extracellular solute-binding protein | GMCHHGAN_003850 | Overabundant by 125.16× | 0.000358 |
| Extracellular solute-binding protein | GMCHHGAN_003842 | Overabundant by 11.61× | 0.027 |
| Substrate-binding domain-containing protein (transcription factor) | GMCHHGAN_003853 | Overabundant by 9.92× | 0.00031 |
| Hydroxyacid dehydrogenase | GMCHHGAN_003851 | Only in xanthan condition | 0.000012 |
| FAD-dependent oxidoreductase | GMCHHGAN_003843 | Overabundant by 26.24× | 0.0018 |

The carbohydrate ABC transporter permease (GMCHHGAN_003840) and the LacI family DNA-binding transcriptional regulator (GMCHHGAN_003844) were not detected.

At the end-growth point of OD_600_ of greater than 2.7, 11 out of the 15 proteins detected at any point within the experiment in the XUR were overabundant in the xanthan condition (73.3%). This means that 11 out of 12 proteins (91.6%) encoded in the XUR which were detected at this time point were significantly overabundant (Supplemental Table 4).

Supplemental Table 4: Proteins encoded within the XUR located on the 147kb pPANIL_6C plasmid which are significantly overabundant in the xanthan condition at an OD_600_ of greater than 2.7

| Protein Name | Accession | Fold Change / Detection | p-Value |
| --- | --- | --- | --- |
| Polysaccharide lyase 8 family protein | GMCHHGAN_003836 | Overabundant by 41.87× | 0.044 |
| α-mannosidase GH38 | GMCHHGAN_003845 | Overabundant by 550.17× | 0.012 |
| Glycoside hydrolase family 3 protein | GMCHHGAN_003847 | Overabundant by 79.82× | 0.034 |
| Carbohydrate ABC transporter permease | GMCHHGAN_003848 | Only in xanthan condition | 0.00000244 |
| Carbohydrate ABC transporter permease | GMCHHGAN_003849 | Only in xanthan condition | 0.0000161 |
| Extracellular solute-binding protein | GMCHHGAN_003842 | Overabundant by 7.93× | 0.00239 |
| Extracellular solute-binding protein | GMCHHGAN_003850 | Overabundant by 34.36× | 0.0086 |
| Substrate-binding domain-containing protein (transcription factor) | GMCHHGAN_003853 | Overabundant by 3.79× | 0.00089 |
| LacI family DNA-binding transcriptional regulator | GMCHHGAN_003839 | Overabundant by 1.49× | 0.025 |
| Hydroxyacid dehydrogenase | GMCHHGAN_003851 | Only in xanthan condition | 0.0000275 |
| FAD-dependent oxidoreductase | GMCHHGAN_003843 | Overabundant by 5.98× | 0.00511 |
